# Maternal breast milk microbiota and immune markers in relation to subsequent development of celiac disease in offspring

**DOI:** 10.1038/s41598-022-10679-x

**Published:** 2022-04-22

**Authors:** Jelena Štšepetova, Kärt Simre, Aili Tagoma, Oivi Uibo, Aleksandr Peet, Heli Siljander, Vallo Tillmann, Mikael Knip, Reet Mändar, Raivo Uibo

**Affiliations:** 1 Department of Microbiology, Institute of Biomedicine and Translational Medicine, University of Tartu, Ravila Street 19, 50411 Tartu, Estonia; 2 Department of Immunology, Institute of Biomedicine and Translational Medicine, University of Tartu, Ravila 19, 50411 Tartu, Estonia; 3 Children’s Clinic, Tartu University Hospital, Lunini 6, 50406 Tartu, Estonia; 4 Department of Pediatrics, Institute of Clinical Medicine, University of Tartu, Lunini 6, 50406 Tartu, Estonia; 5 Pediatric Research Center, Children’s Hospital, University of Helsinki, Helsinki University Hospital, Stenbäckinkatu 9, PO Box 347, Helsinki, Finland; 6 Research Program for Clinical and Molecular Metabolism, Faculty of Medicine, University of Helsinki, Biomedicum 1, Haartmaninkatu 8, 00290 Helsinki, Finland; 7 Center of Military Medicine, Finnish Defence Forces Logistics Command, Tampere, Finland; 8 Center for Child Health Research, Tampere University Hospital, Teiskontie 35, 33520 Tampere, Finland; 9 Competence Center on Health Technologies, Teaduspargi 13, 50411 Tartu, Estonia

**Keywords:** Bacteria, Pathogenesis

## Abstract

The potential impact of the composition of maternal breast milk is poorly known in children who develop celiac disease (CD). The aim of our study was to compare the microbiota composition and the concentrations of immune markers in breast milk from mothers whose offspring carried the genetic predisposition to CD, and whether they did or did not develop CD during follow-up for the first 3 years of life. Maternal breast milk samples [CD children (n = 6) and healthy children (n = 18)] were collected 3 months after delivery. Enzyme-linked immunosorbent assays were used to measure TGF-β1, TGF-β2, sIgA, MFG-E8 and sCD14. For microbiota analysis, next generation (Illumina) sequencing, real-time PCR and denaturing gradient gel electrophoresis were used. Phylotype abundance and the Shannon ‘H’ diversity index were significantly higher in breast milk samples in the CD group. There was higher prevalence of the phyla *Bacteroidetes* and *Fusobacteria*, the classes *Clostridia* and *Fusobacteriia*, and the genera *Leptotrichia*, *Anaerococcus*, *Sphingomonas*, *Actynomyces* and *Akkermansia* in the CD group. The immunological markers were differently associated with some Gram-negative bacterial genera and species (*Chryseobacterium*, *Sphingobium*) as well as Gram-positive species (*Lactobacillus* *reuteri*, *Bifidobacterium* *animalis*). In conclusion, the microbiota in breast milk from mothers of genetically predisposed offspring who presented CD showed a higher bacterial phylotype abundance and diversity, as well as a different bacterial composition, as compared with the mothers of unaffected offspring. These immune markers showed some associations with bacterial composition and may influence the risk for development of CD beyond early childhood.

## Introduction

Celiac disease (CD) is a systemic immune-mediated disorder triggered by gluten and related prolamins in genetically susceptible individuals^1,2^. This disease is characterized by a variable combination of gluten-dependent clinical manifestations, CD-specific antibodies, HLA-DQ2/8 haplotypes and enteropathy^1–3^.

Previously, it has been demonstrated that breast feeding, its duration and the introduction of gluten into an infant’s diet may be determinants in the pathogenesis of CD^4–6^. Breast milk components promote oral tolerance to dietary antigens by modulating immune development and infant gut microbiota composition^4,7–10^. The latter is also thought to be involved in the pathogenesis of CD^11^. In contrast, some studies have reported no effect of breast-feeding on the development CD in children^12,13^.

Earlier work has shown that infants with an increased genetic risk for CD were colonized with a lower proportion of *Actinobacteria* and a higher proportion of *Firmicutes* and *Proteobacteria* than infants with a low genetic risk for the development of CD^6^. De Palma et al*.* demonstrated that the type of milk feeding in relation to HLA-genotype played a role in establishing infant gut microbiota^14^. The HLA-DQ genotype may specifically influence the colonization process of *Bacteroides* species^3,15^. In addition, it has been observed that breast milk from CD mothers have lower levels of TGF-β1, sIgA and a reduced abundance of *Bifidobacterium* sp. and *B.* *fragilis* compared with healthy women^5^.

As the frequency of CD has increased we hypothesized that the immunological composition and microbiota of breast milk might differ between the mothers whose offspring carry a genetic susceptibility to CD, and developed CD relative to those who remain unaffected. The current study set out to characterize breast milk microbiota composition and its possible relationship with immunological markers (TGF-β1, TGF-β2, sIgA, MFG-E8, and sCD14) in mothers whose offspring presented or did not present CD during the first 3 years of life.

## Results

### Immune markers in breast milk from mothers of CD and control offspring

There were no significant differences between TGF-β1, TGF-β2, sIgA, MFG-E8, and sCD14 levels in the breast milk from mothers in the CD group and the breast milk from mothers in the control group (Table 1).

**Table 1:** Immune markers in the breast milk from mothers of celiac disease (CD) and control children (median, quartiles Q1, Q3).

Immune marker	CD group	Control group	*p-*value
TGF-β1 (pg/ml)	102.3 (15, 229.6)	157.5 (59.73, 270.0)	0.42
TGF-β2 (pg/ml)	1603 (1170, 3516)	1817 (1176, 2463)	0.97
sIgA (mg/ml)	0.69 (0.05, 1.68)	0.80 (0.05, 1.45)	0.82
MFG-E8 (mg/ml)	0.014 (0.009, 0.027)	0.013 (0.008, 0.017)	0.69
sCD14 (pg/ml)	232,200 (46,940, 1,118,000)	323,900 (0, 1,285,000)	0.92

### The microbiome of the maternal breast milk

We applied Illumina sequencing of the 16S *r*RNA V4 region to reveal the full microbiome of these investigated samples. A total of 6,475,320 high quality reads were obtained, or 269,805 ± 239,377 reads per milk sample. The OTUs were classified into known taxa (7 phyla, 17 classes, 44 genera, and more than 90 species) and unclassified groups.

Phylotype abundance and the Shannon ‘H’ diversity index were significantly higher in milk samples from the mothers in the CD group than in the maternal samples in the control group (*p* = 0.016; *p* = 0.008, respectively) (Table 2).

**Table 2:** Average number (± SD) of sequences, phylotype abundance (OTUs) and Shannon ‘H’ diversity index in the samples analyzed (^*^p = 0.016; ^**^p = 0.008).

Samples	Number of reads	Phylotype abundance (OTUs)	Shannon ‘H’ index (diversity)
CD group	334,403 ± 410,283	276.6 ± 28.6*	3.80 ± 0.41**
Control group	248,272 ± 161,407	219.5 ± 65.6*	2.88 ± 1.03**

A principal coordinate analysis (PCoA) plot based on different taxonomic levels (phylum, class and genus) was generated to assess the relationships between the community structures of these samples. Phylum and class abundance data indicated significant inter-individual variability (Fig. S1A,B), while the PCoA plot of relative genus abundance demonstrated weighted clustering in the CD group (Fig. S1C).

### Phyla

**Figure 1:**
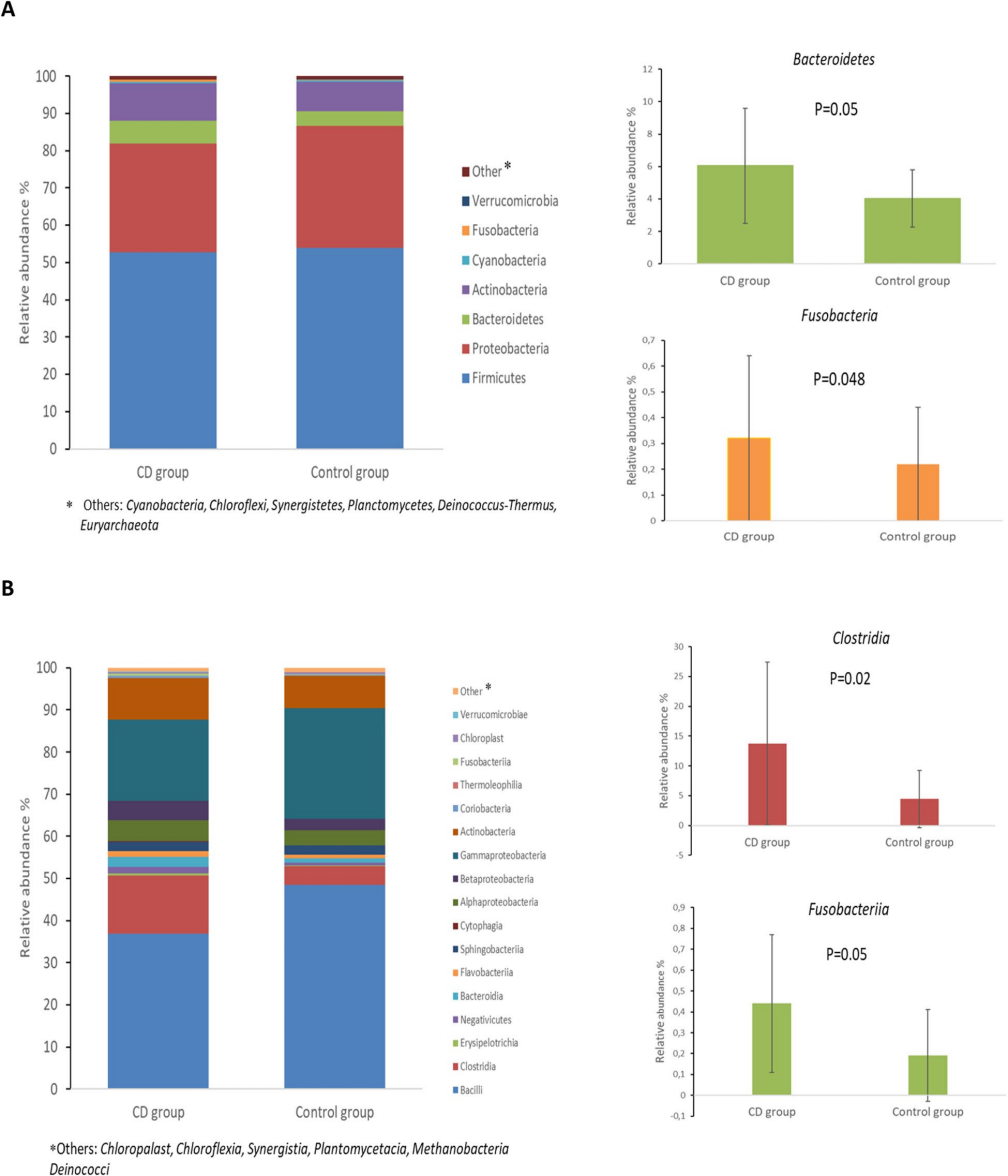

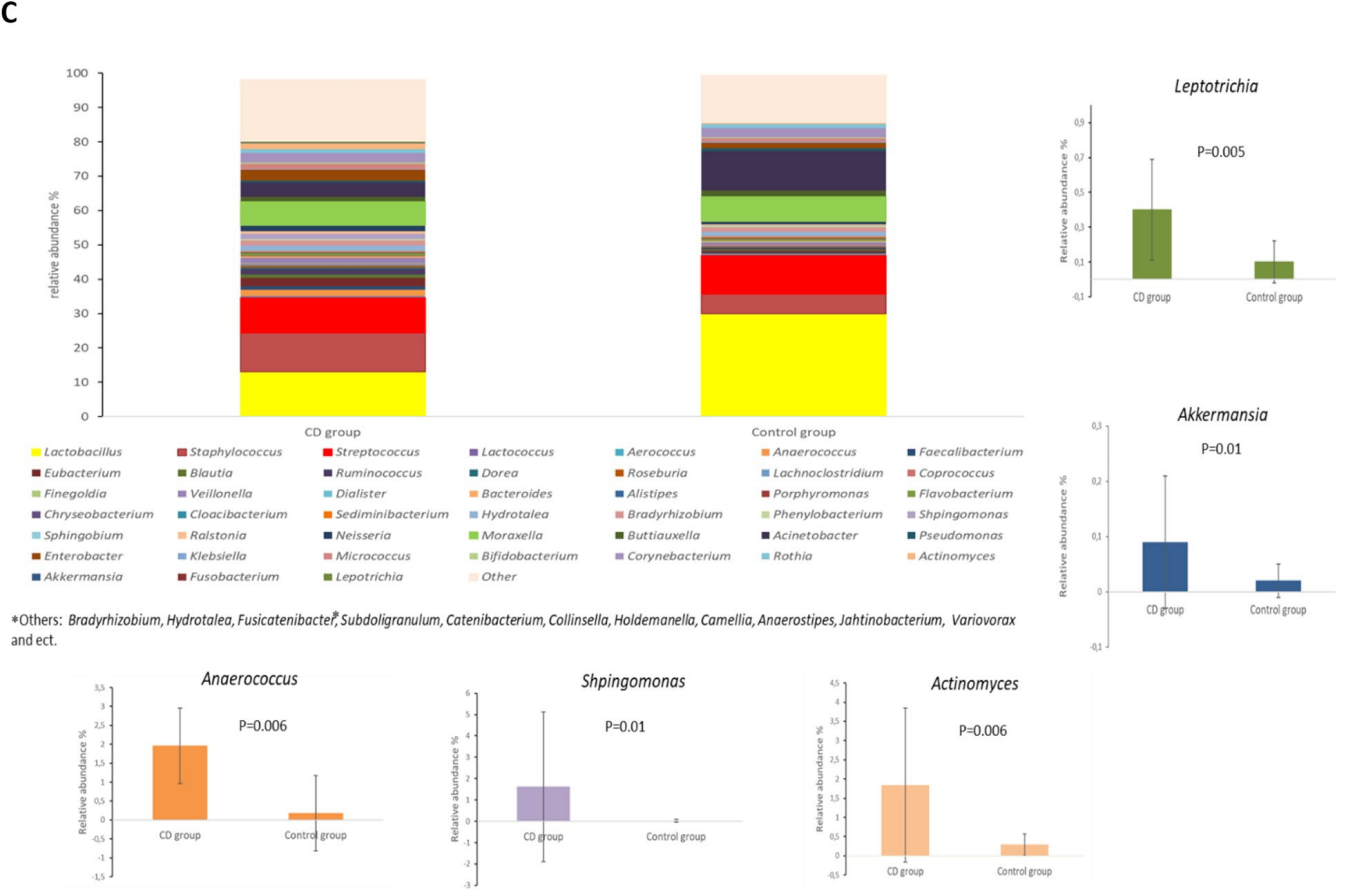
The relative abundance (median) of most frequent **(A)** phyla, **(B)** classes and **(C)** genera of microbial communities in maternal breast milk samples [coeliac disease (CD) versus control group]. Statistically significant relative abundances (mean ± SD) based on different taxonomic levels: phyla (*Bacteroidetes,* *Fusobacteria*); classes (*Clostridia,* *Fusobacteriia*) and genera (*Leptotrichia,* *Akkermansia,* *Anaerococcus,* *Shpingomonas* and *Actinomyces*).

In both study groups, the phylum *Firmicutes* displayed the highest relative abundance (medians of 48.9% and 60.2% for the CD group and the control group, respectively) (Fig. 1A, Supplementary Table S2). In addition, *Proteobacteria* and *Actinobacteria* were also quite abundant (median 29.0% and 9.8% for the CD group *vs*. 23.6% and 8.1% for the control group). A borderline statistical significance was detected for the relative abundance of *Bacteroidetes* and *Fusobacterium* phyla (*p* = 0.056 and *p* = 0.048; Fig. 1A, Supplementary Table S2).

### Classes

At the class level, *Bacilli* displayed the highest relative abundance both in the CD group (median 30.9%) and in the control group (median 56.1%), followed by *Gammaproteobacteria* (20.8% and 14.5%), *Actinobacteria* (8.9% and 8%) and *Alphaproteobacteria* (4% and 3.7%) (Fig. 1B, Supplementary Table S3). Statistically significant differences were only found for the relative abundance of class *Clostridium* (*p* = 0.02) and *Fusobacteriia* (*p* = 0.05) (Fig. 1B, Supplementary Table S3).

### Genera

The most abundant genera of bacteria both in the CD and the control groups was *Lactobacillus* (median 10.3% and 34%, respectively), followed by *Streptococcus* (10.9% and 7.5%), *Staphylococcus* (4.9% and 4.9%), *Buttiauxella* *(*4.8% and 4.4%), and *Rothia* (2.8% and 2.6%) (Fig. 1C, Supplementary Table S4). The prevalence of the genera *Anaerococcus* (*p* = 0.006), *Shpingomonas* (*p* = 0.01), *Actinomyces* (*p* = 0.006), *Leptotrichia* (*p* = 0.005), and *Akkermansia* (*p* = 0.01) were significantly higher in the CD group (Supplementary Table S4). Moreover, the relative abundances of *Sphingomonas* (*p* = 0.04) and *Akkermansia* (*p* = 0.05) were significantly higher in the CD group (Fig. 1C, Supplementary Table S4).

### Species

The most abundant species in the CD group in comparison to the control group were *Actinomyces* *odontolyticus* (*p* = 0.03), *Anaerococcus* *hydrogenalis* (*p* = 0.04), and *A.* *octavius* (*p* = 0.01) (Supplementary Table S5). The prevalence of *A.* *muciniphilia* was also increased in the CD group (*p* = 0.002). It is noteworthy that the milk samples in the control group had lower relative abundance of *Faecalibacterium* *prausnitzii* (*p* = 0.049) (Supplementary Table S5), while unclassified *Leptotrichia* and *Akkermansia* were detected only in the CD group. *Lactobacillus* *salivarius* (83 and 78%) and *Bifidobacterium* *animalis* (both 50%) were the most prevalent species among lactic acid bacteria in both study groups, although their mean counts were less than 1% (Supplementary Table S5).

**Figure 2:**
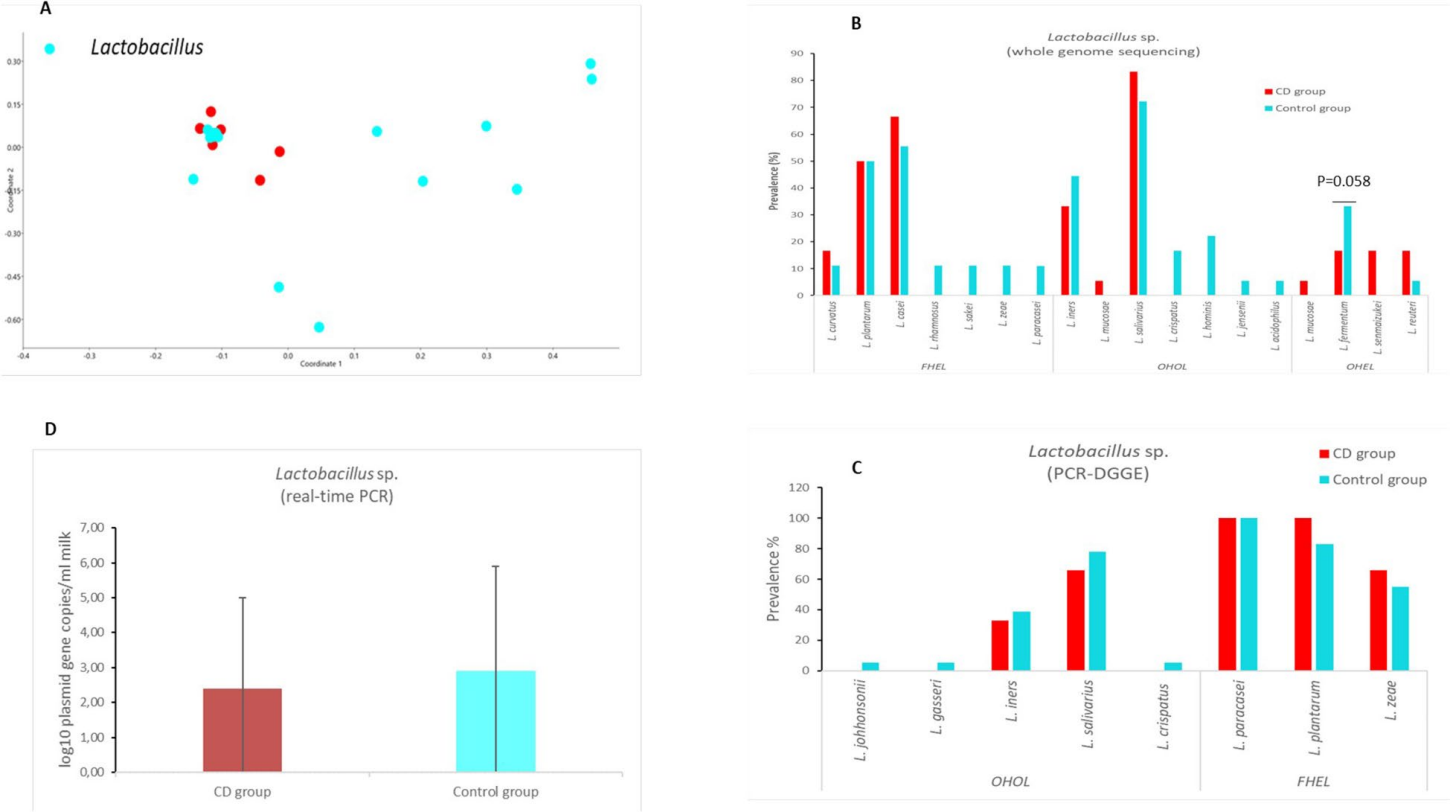
(**A**) Principal coordinate analysis (PCoA) of lactobacilli communities in studied samples. The plot demonstrates different clustering of different breast milk specimens [CD group (red) versus control group (blue)]. Prevalence (%) of different *Lactobacillus* sp. in breast milk samples according to whole genome sequencing **(B)** and denaturing gradient gelelectrophoresis **(C)** methods. *FHEL* facultative heterofermentative lactobacilli, *OHOL* obligate homofermentative lactobacilli, *OHEL* obligate heterofermentative lactobacilli. **(D)** Total counts (log10plasmid gene copies/ml milk) of *Lactobacillus* sp. in breast milk samples of CD and control groups by real-time PCR (mean ± SD).

**Figure 3:**
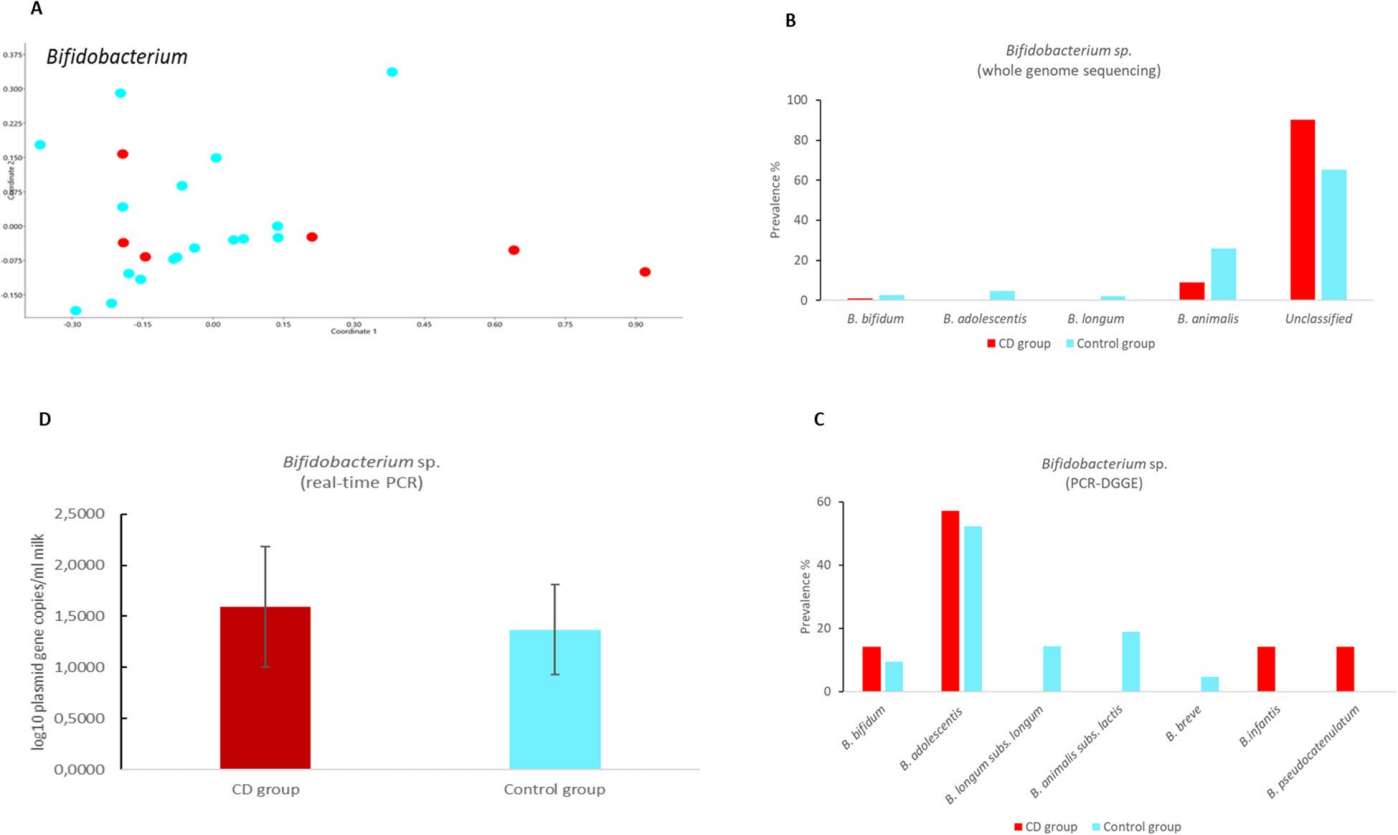
**(A)** Principal coordinate analysis (PCoA) of bifidobacteria communities in the samples analysed. The plot demonstrates clustering of different breast milk specimens [CD group (red) versus control group (blue)]. Prevalence (%) of different *Bifidobacterium* sp. in breast milk samples according to whole genome sequencing **(B)** and denaturing gradient gel-electrophoresis **(C)** methods. **(D)** Total counts (log10plasmid gene copies/ml milk) of *Bifidobacterium* sp. in breast milk samples of CD and control groups by real-time PCR (mean ± SD).

A total of nineteen *Lactobacillus* species (*L.* *plantarum*, *L.* *curvatus*, *L.* *iners,* *L.* *mucosae*, *L.* *casei,* *L.* *fermentum,* *L.* *salivarius,* *L.* *reuteri,* *L.* *rhamnosus,* *L.* *crispatus,* *L.* *hominis,* *L.* *sakei,* *L.* *zeae,* *L.* *jensenii,* *L.* *senmaizukei,* *L.* *paracasei,* *L.* *oligofermentas,* *L.* *gasseri,* *and* *L.* *acidophilus*) (Fig. 2B) and four species of *Bifidobacterium* (*B.* *bifidum*, *B.* *animalis*, *B.* *adolescentis*, and *B.* *longum*) were detected by whole genome sequencing (Fig. 3B). Additionally, *L.* *johhansonii* (Fig. 2C) and *B.* *pseudocatenulatum,* *B.* *infantis* *and* *B.* *breve* were using denaturing gradient gel electrophoresis (DGGE) (Fig. 3C, Table S9).

A principal coordinate analysis (PCoA) plot of relative abundance data of *Lactobacillus* and *Bifidobacterium* species indicated quite different results, with weighted clustering of *Lactobacillus* in the CD group (Fig. 2A) and *Bifidobacterium* in the control group (Fig. 3A). No differences were found between the groups in quantitative counts for *Lactobacillus* or *Bifidobacterium* (Figs. 2D, 3D; Table S9). Milk samples in the CD group showed borderline significance for the lower relative abundance of *L.* *fermentum* when compared to the control group (*p* = 0.058, Supplementary Table S5).

### Associations between breast milk microbiota and immune markers

*A.* *muciniphila* showed a positive association with TGF-β1 (*p* = 0.04) and TGF-β2 (*p* = 0.007) (Table 3). Higher counts of the genera *Chryseobacterium* (*p* = 0.04) and *Sphingobium* (*p* = 0.01) were seen in milk samples with lower TGF-β2 levels. MFG-E8 correlated positively with the bacterial classes *Flavobacteriia* (*p* = 0.03) and *Bacilli* (*p* = 0.05) and inversely with the species *B.* *animalis* (*p* = 0.03)*.* In addition, the species *L.* *reuteri* was positively (*p* = 0.04) and *B.* *animalis* was inversely (*p* = 0.02) correlated with sCD14 levels (Table 3). All the correlation coefficients are presented in Supplementary Tables S6–S8.

**Table 3:** Spearman’s rank-order correlations between bacteria present in human milk and immune markers**.**

Bacteria	Immune marker	R^2^	*p*-value
*A.* *muciniphila* (species)	TGF-β1	0.43	0.04
*Chryseobacterium* (genus)	TGF-β2	−0.42	0.04
*Sphingobium* (genus)		−0.51	0.01
*A.* *muciniphila* (species)		0.54	0.007
*Flavobacteriia* (class)	MFG-E8	0.45	0.03
*Bacilli* (class)		0.40	0.05
*B.* *animalis* (species)		−0.44	0.03
*L.* *reuteri* (species)	sCD14	0.43	0.04
*B.* *animalis* (species)		−0.47	0.02

## Discussion

In this case–control study, we wanted to identify whether there were differences in the immunological composition and microbiota of breast milk between mothers whose genetically predisposed offspring developed CD during the first 3 years of life or remained unaffected. To the best of our knowledge, the present study was the first where maternal breast milk samples were analyzed for both immunological markers and for microbiota composition.

We demonstrated that, compared to milk samples from mothers of unaffected offspring, the human milk microbiota in the mothers in the CD group had a higher phylotype abundance and diversity, with a higher abundance of the phyla *Bacteroidetes* and *Fusobacteria*, the classes *Clostridia* and *Fusobacteria*, as well as the genera *Leptotrichia*, *Anaerococcus*, *Sphingomonas*, *Actinomyces,* and *Akkermansia*. Increased relative abundance for species such as *A.* *odontolyticus*, *A.* *hydrogenalis*, *A.* *octavius*, and *F.* *prausnitzii,* and decreased abundance for *L.* *fermentum* were also observed in the CD group. We observed an association between certain immune markers (TGF-β1, TGF-β2, MFG-E8, and sCD14) and some Gram-negative (*Chryseobacterium*, *Sphingobium,* and *A.* *muciniphila*) and Gram-positive bacteria (*L.* *reuteri* and *B.* *animalis)*.

Breast milk does not contain abundant culturable microbiota. However, some next generation sequencing studies have revealed *Streptococcus,* *Staphylococcus,* *Gemella,* *Veillonella,* *Rothia,* *Lactobacillus,* *Propionibacterium,* *Corynebacterium,* and *Pseudomonas* in milk samples from healthy woman^8,16^. In the current study, the most abundant genus in all milk samples was *Lactobacillus,* followed by *Streptococcus,* *Staphylococcus,* *Moraxella,* *Acinetobacter,* *Enterobacter,* and *Corynebacterium*. Some studies have indicated that the primary bacterial taxa in milk may vary across population, suggesting that geographic, genetic, and dietary factors could be influencing microbial diversity in breast milk. In Finland, *Leuconostoc,* *Weisella,* and *Lactococcus* were the most predominant genera in milk samples^17^, while in Mexican–American mothers the predominance of *Streptococcus,* *Staphylococcus,* *Xanthomonadaceae,* and *Sediminibacterium* was observed^18^.

Breast milk is a major source of bacterial diversity for the neonatal gut, including gut-associated obligate anaerobes, and therefore significantly influences gut colonization and the maturation of the immune system^6,19^. Different genetic and epigenetic factors may influence host microbiome interactions and shift the immune balance in subjects with CD^20^. In case of the intestinal microbiome, higher microbial richness and diversity values are considered to be more health-supporting^21^. However, this may not be true in case of some other biotopes (for example, the reproductive tract)^22^. Quite unexpectedly, we found that samples taken from mothers whose children developed CD had a higher phylotype abundance and Shannon diversity index than healthy samples. It is interesting that a recent study by Huang et al. also demonstrated a significantly higher alfa diversity in fecal samples from CD progressors compared to healthy controls at an age of one year^23^. This indicates that the increase of a few dominant bacteria would be expected in cases of infection and suggests that milk microbiota is not activating an immune response in the host. Unfortunately, in the current study inflammatory markers were not measured. In contrast, it has been shown that in case of allergy, feeding breast milk with lower bacterial diversity during the first month of life was related to an increased risk of allergy in childhood^24^. We cannot rule out the influence of anticommensal antibodies on microbiota composition, which may differ even between healthy individuals and may have an impact on microbiome composition^25,26^.

Olivares et al.^6^ showed that the breast milk of mothers with CD demonstrated significant reductions in the counts of *Bifidobacterium* spp. and *Bacteroides* *fragilis* in comparison to healthy mothers. In the present study we observed a significantly increased relative abundance of class *Clostridia* in the milk samples from the CD group. We also found differences in the relative abundance of the genus *Anaerococcus* (incl. *A.* *hydrogenalis*, *A.* *octavius*) and *F.* *prausnitzii*. The presence of *A.* *octavius* was reported previously in breast milk samples from healthy Spanish women. In the same study, an association was shown between the amounts of proteins in milk and *Anaerococcus*^27^. *F.* *prausnitzii* is the major butyrate producer in the gastrointestinal tract. It has anti-inflammatory properties important for gastrointestinal microbiota homeostasis and is associated with a range of metabolic processes in the human mucosa^28^. A Spanish study showed that the adherence to a gluten-free diet resulted in slight microbiota shifts in adults, including a decreased abundance of *F.* *prausnitzii*^29^. As *F.* *prausnitzii* produces the anti-inflammatory short-chain fatty acid butyrate^30,31^, this species may have a key role in maintaining the integrity and function of the mucus barrier, representing an important aberrant mechanism in the development of CD.

We also found that the prevalence of the genus *Akkermansia* was higher in the CD group samples. Interestingly, unclassified *Akkermansia* species were found only in that group. A study by Huang et al. observed the *Akkermansia* genus only in the control group and not in CD progressors^23^. However, they analyzed fecal samples and not breast milk samples. In contrast, some experiments in mice have demonstrated that a gluten-free diet can induce changes in the intestinal microbiota by increasing the number of *Akkermansia*^32,33^. It can be speculated that this species may translocate from its normal habitat (intestine) to an unusual habitat (breast) in cases of CD.

An increased relative abundance for the genus *Actinomyces* and for a species of this genus, *A.* *odontolyticus,* were observed in the CD group. Mucosal membranes of the oropharynx, gastrointestinal tract and female genital tract represent the normal habitat of *Actinomyces*^34^. They have also been observed to associate with breast infection^35,36^. Although being low in virulence, they may rely on the presence of co-pathogens, such as anaerobic bacteria, to enhance pathogenicity^37^. Previously, *A.* *graevenitzii* was identified in biopsy specimens from the proximal small intestine of children with CD, and an unidentified *Actinomyces* sp. was shown to be attached to the epithelial lining^38^.

For detection of *Lactobacillus* and *Bifidobacterium*, we applied two methods, next generation sequencing and PCR-DGGE, since Smidt et al*.,* demonstrated the partial concordance of different molecular methods for the detection of lactic acid bacteria^39^. In our study, PCR-DGGE appeared to be more sensitive for the identification of *Bifidobacterium* sp., while more than 80% of *Bifidobacterium* sp. detected by Illumina sequencing were unclassified.

In line with our findings, other studies have also shown the presence of *Lactobacillus* sp. and *Bifidobacterium* sp. in breast milk^6,40^. A low relative abundance of *L.* *fermentum* was observed in breast milk in the CD group. The expected pathway by which lactobacilli may get into milk is entero-mammary transport through dendritic cells^41^. Production of short-chain fatty acids by lactobacilli strains may play a role in the pathogenesis of CD. *L.* *fermentum* produces both lactic and succinic acids, which in turn modulate the key functions of many main players in the innate immune response^42^. They mediate macrophage dependent anti-inflammatory effects^43^ and possess antioxidative capacities^44^. Additionally, it was demonstrated that *L.* *fermentum* isolated from breast milk may have an immunostimulatory effect by inducing proinflammatory cytokines in rodent bone marrow derived macrophages^40^. In vivo assays in mice revealed that the ingestion of *L.* *fermentum* enhanced the production of Th1 cytokines by spleen cells and increased the IgA concentration in feces^45^. Likewise, *Lactobacillus* strains have high amylase trypsin inhibitor-metabolizing capacity to ameliorate many adverse effects induced by wheat immunogenic proteins^46^. Accordingly, one may assume that *L.* *fermentum* might protect against the development of CD.

There were no significant differences in the levels of the immunological markers analyzed between the CD and the control group, but some significant associations with milk microbiota were observed. The presence of *Chryseobacterium* and *Sphingobacterium* (both being Gram negative environmental bacilli) correlated inversely with breast milk TGF- β2 levels, while *A.* *muciniphila* (anaerobic mucin-degrading bacterium) showed a positive correlation with both TGF- β1 and TGF- β2. TGF-β belongs to a growth factor family that is responsible for the development and maturation of the mucosal immune system^47^. In our study, we also observed that higher counts of *L.* *reuteri* and lower counts of *B.* *animalis* in breast milk were associated with higher sCD14 concentrations. The latter bacterial species were also associated with higher concentrations of MFG-E8. Vidal et al*.* demonstrated that milk sCD14 acts as a “sentinel” molecule and immune modulator in homeostasis and in the defense of the neonatal intestine^48^. Supplementation of maternal breast milk with the probiotic *L.* *reuteri* increased the TGF-β2 concentration level in breast milk and decreased sensitization in breast-fed infants^49^. In addition, the *Bacilli* and *Flavobacteriia* classes correlated positively with the concentration of MFG-E8, which has been shown to have antiviral effects and to protect against rotavirus both in a cultured cell model as well as in infants^50–52^. Furthermore, it prevented the tissue damage caused by prolonged inflammation by clearing apoptotic cells and thereby facilitating immune resolution^53^.

Our study had many strengths. All study subjects in both countries were monitored and diagnosed with identical protocols. All CD diagnoses were confirmed by a biopsy of the small intestine. Although patients came from two different countries, all the analyses were carried out in one laboratory. Two different methods in parallel were applied for investigating *Lactobacillus* sp. and *Bifidobacteria* sp. in breast milk microbiota.

Our study also had some limitations. The number of cases was quite small, limiting the statistical power of this study. The mothers in the CD group were younger than the mothers in the control group. When interpreting the results this may be important because it is known that maternal age may influence milk composition and microbiota^54^. The children were observed for only 3 years and we lack information about possible new CD cases beyond that age. As we analyzed only a small number of breast milk samples taken at the age of 3 months, larger longitudinal studies are needed for a better understanding of possible differences between these two groups.

In conclusion, this study provides new insights into the complex breast milk microbiota composition in mothers whose offspring carry a genetic susceptibility to CD but have different disease outcomes. The breast-milk microbiota of mothers of children who developed CD differed in terms of higher bacterial phylotype abundance and diversity, as well as in relation to bacterial composition when compared to the mothers of unaffected children. The immunological markers were differently associated with bacterial composition and could also influence the risk of development CD in later life. Future studies are needed to reveal whether differences in breast milk composition ultimately influence the development of CD.

## Materials and methods

### Study subjects

Participants in the current analysis were recruited from the DIABIMMUNE Study, which was originally carried out in Estonia, Finland, and Russian Karelia from September 2008 to October 2013. The exact recruitment criteria and number of participants have been described previously^55^. For the current analysis, samples and data from Estonia and Finland were used.

This study was conducted in accordance with the Declaration of Helsinki and local ethical committees respectively approved this study (Ethics Review Committee on Human Research of the University of Tartu in Estonia and Ethical Committee for Psychiatric Diseases and Diseases in Children and Adolescents, Helsinki and Uusimaa Hospital District in Finland). Written informed consent was obtained from all parents for the participation of their child in this study.

Children who were followed from birth to the age of 3 years and those who later developed CD (n = 6) were included in our analysis. The diagnosis of CD was confirmed according to the European Society for Pediatric Gastroenterology, Hepatology and Nutrition^2^, including the abnormal morphology of small intestinal biopsy in accordance with the Marsh classification modified by Oberhuber^56^. From the same DIABIMMUNE cohort we selected 18 controls based on the CD-specific HLA DR/DQ genotype, country of birth, time of birth and sex of each patient.

Five children in the CD group were exclusively breastfed, while one child was mostly bottle-fed during the time of breast milk collection. The corresponding numbers in the control group were 15 and 3. One CD patient and his controls were from Estonia, while all the others were from Finland. The proportions of boys and girls were equal in the CD and control groups. All offspring were HLA DQ 2.5 positive (two CD patients were HLA DQ 2.5 homozygotes). Each infant was full-term (gestational age 37–42 weeks) and born as a singleton. None of the mothers or fathers of the offspring had CD. The mean age was 2.4 years (range 1.5–3.0 years) at seroconversion to positivity for IgA- class tissue transglutaminase antibodies in the CD group. General characteristics of the CD and the control group children are summarized in Table 4.

**Table 4:** General characteristics of the celiac disease (CD) group and the control group (*—data missing for one child; **p = 0.002).

Characteristics	CD group (n = 6)	Control group (n = 18)
**Sex**		
Male (%)	3 (50%)	9 (50%)
Female (%)	3 (50%)	9 (50%)
Birth weight (g) mean, (range)	3703 (3200–4075)	3661 (2700–4530)
Birth length (cm) mean, (range)	50.8 (48–53.5)	51.3 (48–54.5)*
**Offspring HLA**		
HLA DQ 2.5 homozygotes (%)	2 (33%)	4 (22%)
HLA DQ 2.5 (%)	4 (67%)	14 (78%)
**Mode of delivery**		
Vaginal (%)	6 (100%)	15 (83%)
Caesarean (%)	0	3 (17%)
Mean maternal age in years at delivery (range)	26 (23–30)**	32.8 (23–42)**

### Collection and processing of samples

Breast milk samples were collected from mothers in both groups 3 months after delivery. The milk samples were obtained by manual expression into sterile bags. Samples were immediately put into a −20 ºC freezer and further stored at −80 ºC until processing. Breast milk samples were thawed and centrifuged for 15 min at 1000*g* at 4 ºC and after that the aqueous fraction was collected. The latter was used for further analyses. Centrifugation was repeated twice.

All breast milk analyses were carried out in the Department of Immunology and Department of Microbiology at Institute of Biomedicine and Translational Medicine, University of Tartu, Estonia.

### Immune markers in breast milk

We used enzyme-linked immunosorbent assay (ELISA) to measure TGF-β1, TGF-β2, sIgA, MFG-E8 (lactadherin), and sCD14. For measuring TGF-β1, TGF-β2, MFG-E8, and sCD14 we used commercial kits from R&D Systems (Minneapolis, MN, USA) according to the manufacturer’s instructions. For sIgA, we used a commercial kit from Bethyl Laboratories (Montgomery, AL, USA) according to the manufacturer’s instructions.

### Molecular analyses

#### DNA extraction

Bacterial DNA from human milk was extracted using the PureLink™ Microbiome DNA Purification Kit [Invitrogen, using an ELMI SkyLine instrument (ELMI Ltd., Riga, Latvia)]. Human milk samples were centrifuged at 4000*g* for 30 min, after which the supernatant was removed. Cell pellets were washed with phosphate-buffered saline, centrifuged at 13,000*g* × 3 min at room temperature and the pellets were resuspended in a kit-specific lysis buffer. The protocol was then continued as described by the manufacturer (Invitrogen, USA).

#### Illumina sequencing

16S library preparation was performed using an in-house sequencing protocol with the V4 (F515/R806) primer pair^57^. Sequencing was performed using an iSeq 100 (SN FS10000643, Illumina) and an iSeq 100 i1 Reagent kit (Illumina) in the 2 × 150 bp mode and using the dual index setup with two-read sequencing protocol. 16S rDNA sequence data were analyzed using BION-meta (https://www.box.com/bion), according to the author’s instructions and in-house scripts. First, sequences were cleaned at both ends using a 99.5% minimum quality threshold for at least 18 of 20 bases for the 5′-end and 28 of 30 bases for 3′-end. The reads were then joined, followed by the removal of read pairs shorter than 150 bp. Then, sequences were cleaned from chimeras and clustered using a 95% oligonucleotide similarity (k-mer length of 8 bp and a step size of 2 bp). Lastly, consensus reads were aligned to the SILVA reference 16S rDNA database (v123) using a word length of eight and similarity cut-off of 90%. The bacterial designation was analyzed at different taxonomic levels down to the species when applicable.

#### Primers and probes

Denaturing gradient gel electrophoresis, sequencing of DGGE amplicons and real-time PCR were performed with primers and probes listed in supplementary Table S1^58–61^.

#### Real-time PCR

In order to establish a quantitative assay, plasmid standards were generated using the method described in Bartosch et al*.*^62^. The amplified 16S rRNA gene region (amplified with specific primers) from *B.* *longum* DSM20219 and *L.* *acidophilus* ATCC4356 was cloned into chemically component *E.* *coli* JM109 cells using the pGEM-T vector system (Promega, Madison, WI, USA). Plasmids were purified with NucleoSpin Plasmid QuickPure kit according to the manufacturer’s instructions (Macherey–Nagel, Düren, Germany). Multiple dilutions of purified plasmids were quantified by spectrophotometry (NanoDrop ND-1000, Thermo Fisher Scientific, Waltham, MA, USA). Quantification of target DNA was achieved by using serial tenfold dilution from 10^5^ to 10^1^ plasmid copies of previously quantified plasmid standards.

Multiplex TaqMan assay PCR reactions were performed in a total volume of 25 µl using TaqMan^®^ Universal PCR Master Mix (Applied Biosystems, Foster City, CA, USA). Each reaction included 5 µl of template DNA, 12.5 µl of TaqMan^®^ Universal PCR Master Mix (Applied Biosystems), 400 nM of (F_allbif_IS and R_alllact_IS primers), 600 nM of (F_alllact_IS and R_alllact_IS primers), 900 nM of (F_Eub and R_Eub primers) and 100 nM both (P_alllact_IS, P_alllbif_IS) and 100 nM (P_Eub) of probes. The real-time PCR conditions consisted of an initial denaturation step at 50 °C for 2 min and then 95 °C for 10 min, continued with an amplification step followed by 40 cycles including denaturation at 95 °C for 15 s, and an annealing-elongation step at 60 °C for 1 min. Amplification and detection of DNA samples by real-time PCR was performed with a 7500 Fast Real-Time PCR System (Applied Biosystems Europe BV, Zug, Switzerland) using optical-grade 96-well plates. Data from triplicate samples were analyzed using Sequence Detection Software version 1.6.3 (Applied Biosystems).

#### Denaturing gradient gel electrophoresis

PCR was performed in a reaction volume of 50 µl containing 25 µl 2× DreamTaq Hot Start PCR Master Mix (Thermo Fisher Scientific), 200 ng of DNA solution and primers at a concentration of 10 µM. The DGGE cycling parameters were 5 min at 94 °C, followed by 35 cycles of 30 s at 57 °C (for primer Im-3 and Im-26), 30 s at 94 °C, 30 s at 62 °C (for primers Bif 164 and Bif 662 + GC), and 45 s at 56 °C (for primer Lac-1 and Lac-2 + GC), and a final extension at 72 °C for 10 min.

The DGGE analysis of PCR amplicons was performed using a Dcode™ System apparatus (Bio-Rad, Hercules, CA, USA). Polyacrylamide gels 8% [wt/vol] involving acrylamide-bisacrylamide [37.5:1] in 0.5× Tris–acetic acid-EDTA buffers with a denaturing gradient was prepared with a gradient mixer and Econopump (Bio-Rad). Gradients from 30 to 60% were employed for the separation of the products amplified with specific primers for *Lactobacillus* spp. and from 45 to 60% for the products amplified with primers specific for *Bifidobacterium* spp.

#### Sequencing of DGGE amplicons

PCR amplicons (Bif164-r and Bif662-f) and (Lac-1 and Lac-2) were purified and concentrated with a QIAquick PCR purification kit (Qiagen, Hilden, Germany) according to the manufacturer’s instructions. Purified amplicons were then cloned into the *E.* *coli* JM 109 strain using the pGEM-T vector system (Promega, Madison, WI, USA). Colonies of ampicillin-resistant transformants were randomly picked from each sample and were subjected to PCR with the pGEM-T specific primers T7 and SP6 (Supplementary Table S1) from lyzed cells to check the size of inserts. Plasmid DNA from selected transformants was isolated using a QIAprep Spin Miniprep kit (Qiagen).

Sequencing reactions were performed using the BigDye Terminator CA v3.1 Cycle Sequencing kit (Applied Biosystem) according to the manufacturer’s instructions. The sequences obtained were analyzed using an automatic LI-COR DNA Sequencer 4000L (Licor, Lincoln, NE, USA) and were corrected manually. All of the sequences were thereafter identified using BLASTN and the NCBI nucleotide database.

### Statistical analysis

Statistical analysis of clinical and molecular data was performed with the R software for Windows 2018 (R: A language and environment for statistical computing. R Foundation for Statistical Computing, Vienna, Austria). The Shapiro–Wilk normality test was used to determine whether the data had a normal distribution. Comparisons between the CD group and the control group were performed using a t-test for normally distributed data and with the Mann–Whitney-Wilcoxon test for data with skewed distribution. To compare the number of positive cases between groups, Fisher’s exact test was used. Correlations were analyzed with a non-parametric Spearman’s rank-order correlation test. Alpha diversity based on the Shannon index of the OTU level. Analysis of beta-diversity of CD and control groups including PCoA of weighted UniFrac distances were performed and visualized using PAST 4.0 version. Statistical significance was accepted as p < 0.05, adjusted for ties.

## Supplementary Information

Supplementary Tables.

Supplementary Figure S1.

## Data Availability

The datasets generated during and/or analysed during the current study are available from the corresponding author on reasonable request.
