# Author Correction: Maternal breast milk microbiota and immune markers in relation to subsequent development of celiac disease in offspring

**DOI:** 10.1038/s41598-022-12277-3

**Published:** 2022-05-12

**Authors:** Jelena Štšepetova, Kärt Simre, Aili Tagoma, Oivi Uibo, Aleksandr Peet, Heli Siljander, Vallo Tillmann, Mikael Knip, Reet Mändar, Raivo Uibo

**Affiliations:** 1 Department of Microbiology, Institute of Biomedicine and Translational Medicine, University of Tartu, Ravila Street 19, 50411 Tartu, Estonia; 2 Department of Immunology, Institute of Biomedicine and Translational Medicine, University of Tartu, Ravila 19, 50411 Tartu, Estonia; 3 Children’s Clinic, Tartu University Hospital, Lunini 6, 50406 Tartu, Estonia; 4 Department of Pediatrics, Institute of Clinical Medicine, University of Tartu, Lunini 6, 50406 Tartu, Estonia; 5 Pediatric Research Center, Children’s Hospital, University of Helsinki, Helsinki University Hospital, Stenbäckinkatu 9, PO Box 347, Helsinki, Finland; 6 Research Program for Clinical and Molecular Metabolism, Faculty of Medicine, University of Helsinki, Biomedicum 1, Haartmaninkatu 8, 00290 Helsinki, Finland; 7 Center of Military Medicine, Finnish Defence Forces Logistics Command, Tampere, Finland; 8 Center for Child Health Research, Tampere University Hospital, Teiskontie 35, 33520 Tampere, Finland; 9 Competence Center on Health Technologies, Teaduspargi 13, 50411 Tartu, Estonia

Correction to: *Scientific Reports* 10.1038/s41598-022-10679-x, published online 22 April 2022

The original version of this Article contained an error in the Acknowledgments section.

“The project was funded by the European Commission (7th Framework Programme, project 202063), the Estonian Research Council (grants nos. IUT20-43; IUT34-19; PRG712; PUT1382; PRG1120), the Estonian Ministry of Education and Research (grant no. KOGU-HUMB), the Academy of Finland (the Centre of Excellence in Molecular Systems Immunology and Physiology Research, 2012–2017, Decision Nos. 250114 and 284597), and Enterprise Estonia (grant no. EU48695). We thank Irja Roots, Kristi Alnek, and Helis Janson for technical assistance.”

now reads:

“The project was funded by the European Commission (7th Framework Programme, project 202063), the Estonian Research Council (grants nos. IUT20-43; IUT34-19; PRG712; PRG1428; PRG1120), the Estonian Ministry of Education and Research (grant no. KOGU-HUMB), the Academy of Finland (the Centre of Excellence in Molecular Systems Immunology and Physiology Research, 2012–2017, Decision Nos. 250114 and 284597), and Enterprise Estonia (grant no. EU48695). We thank Irja Roots, Kristi Alnek, and Helis Janson for technical assistance.”

The original Article has been corrected.

