# Supplementary material for: Maternal breast milk microbiota and immune markers in relation to subsequent development of celiac disease in offspring
Source: Sci Rep. 2022 Apr 22;12:6607. doi: 10.1038/s41598-022-10679-x (PMC9033794; doi:10.1038/s41598-022-10679-x)
Supplement: Supplementary file 1 — Supplementary Tables. [file 41598_2022_10679_MOESM1_ESM.docx]

Supplementary Table S1. Specific primers and probes used for PCR-DGGE (sequencing) and qPCR

| Method | Target groups (amplicon length, T_m,_ assay) | Primers/  Probes | Sequence (5’-3’) | References |
| --- | --- | --- | --- | --- |
| PCR-DGGE | *Lactobacillus* sp.  (496bp, 56°C) | Lac-1  Lac-2  Lac-2+(GC) | GTATTACCGCGGCTGCTGGCAC  ACTCCTACGGGAGGCAGCAGT  **CGCCCGGGGCGCGCCCCGGGCGGG GCGGGGGCACGGGGGG** ACTCCTAC GGGAGGCAGCAGT | Walter et al., 2000 |
|  | *Bifidobacterium* sp.  (1400bp, 57°C)  (498bp, 52°C) | Im3  Im26  Bif164-f  Bif-662-r  Bif-662-r +(GC) | CGGGTGCTICCCACTTTCATG  GATTCTGGCTCAGGATGAACG  GATTTAGGTGACACTATAG  CCACCGTTACACCGGGAA  CGCCCGCCGCGCGCGCGGCGGGCGGGGCGGGGGCACGGGGGGCCACCGTTACACCGGGAA | Satokari et al., 2001 |
| Sequencing | Inserts into pGEM vector | SP6  T7 | TAATACGACTCACTATAGG  GTGAAGCTTACGGT(C/T)TACCTTGTTACGACTT | Promega |
| qPCR | *Lactobacillus* sp. (92bp, 60°C, TaqMan) | F_alllact_IS  R_alllact_IS  P_alllact_IS | TGGATGCCTTGGCACTAGGA  AAATCTCCGGATCAAAGCTTACTTAT  (VIC)-TATTAGTTCCGTCCTTCATC-(NFQ-MGB) | Haarman et al., 2006 |
|  | *Bifidobacterium* sp. (231bp, 60°C, TaqMan) | F_allbif_IS  R_allbif_IS  P_allbif _ IS | GGGATGCTGGTGTGGAAGAGA  TGCTCGCGTCCACTATCCAGT  (VIC)-TCAAACCACCACGCGCCA-(NFQ-MGB) | Haarman et al., 2007 |
|  | All bacteria  (466bp, 59°C, TaqMan) | F_Eub  R_Eub  P_Eub | TCCTACGGGAGGCAGCAGT  GGACTACCAGGGTATCTAATCCTGTT  (FAM)-CGTATTACCGCGGCTGCTGGCAC-(TAMRA) | Haarman et al., 2006 |

The nucleotide probes used in real-time PCR for the detection of the genus *Lactobacillus* and *Bifidobacterium* were labelled with a 5’reporter dye, (VIC), and a 3’quencher, (NFQ-MGB), respectively, and the probe used for the detection of the total counts of bacteria was labelled with a 5’reporter dye, (FAM), and a 3’quencher, (TAMRA), respectively.

Supplementary Table S2. Percentages of positive samples and relative abundance (%) of different bacterial phyla in microbial communities according to sequencing. In case of relative abundance, mean±SD and median (with 1st quartile, 3rd quartile) are presented. *p-value* shows the difference of relative abundance level between the coeliac disease and the control group.

| **Phylum** | **Coeliac disease group** | | **Control group** | | ***p-value*** |
| --- | --- | --- | --- | --- | --- |
|  | % of positive samples | Mean±SD | % of positive samples | Mean±SD |  |
|  |  | Median  (Q1, Q3) |  | Median  (Q1, Q3) |  |
| *Firmicutes* | 100 | 52.80±19.07 | 100 | 53.87±26.17 | 0.92 |
|  |  | 48.94  (38.27, 57.90) |  | 60.20  (37.10, 75.23) |  |
| *Bacteroidetes* | 100 | 6.04±3.55 | 100 | 4.03±1.76 | **0.056** |
|  |  | 5.53  (5.45, 5.97) |  | 3.85  (3.52, 5.05) |  |
| *Proteobacteria* | 100 | 29.08±14.93 | 100 | 32.71±27.16 | 0.82 |
|  |  | 29.03  (21.29, 38.76) |  | 23.60  (12.39, 43.51) |  |
| *Actinobacteria* | 100 | 10.18±4.84 | 100 | 7.85±4.68 | 0.33 |
|  |  | 9.82  (7.81, 13.58) |  | 8.08  (5.62, 9.47) |  |
| *Fusobacteria* | 100 | 0.45±0.32 | 83 | 0.20±0.22 | **0.048** |
|  |  | 0.39  (0.24, 0.60) |  | 0.13  (0.04, 0.25) |  |
| *Cyanobacteria* | 100 | 0.45±0.30 | 94 | 0.47±0.55 | 0.55 |
|  |  | 0.36  (0.28, 0.52) |  | 0.29  (0.09, 0.63) |  |
| *Verrucomicrobia* | 83 | 0.13±0.14 | 83 | 0.08±0.07 | 0.40 |
|  |  | 0.09  (0.06, 0.17) |  | 0.06  (0.01, 0.15) |  |
| *Other* | 100 | 0.87±0.19 | 100 | 0.80±0.54 | 0.10 |
|  |  | 0.91  (0.81, 0.98) |  | 0.70  (0.61, 0.76) |  |

Supplementary Table S3. Percentages of positive samples and relative abundance (%) of different bacterial classes in microbial communities according to sequencing. In case of relative abundance, mean±SD and median (with 1st quartile, 3rd quartile) are presented. *p-value* shows the difference of relative abundance level between the coeliac disease and the control group.

| **Phylum** | **Class** | **Coeliac disease group** | | **Control group** | | **p-value** |
| --- | --- | --- | --- | --- | --- | --- |
|  |  | % of positive samples | Mean±SD | % of positive samples | Mean±SD |  |
|  |  |  | Median  (Q1, Q3) |  | Median  (Q1, Q3) |  |
| *Firmicutes* | *Bacilli* | 100 | 36.86±21.49 | 100 | 48.46±27.61 | 0.31 |
|  |  |  | 30.95  (23.50, 44.55) |  | 56.08  (30.42, 69.60) |  |
|  | *Clostridia* | 100 | 13.78±13.62 | 94 | 4.46±4.81 | **0.02** |
|  |  |  | 9.78  (6.12, 13.79) |  | 3.64  (1.56, 5.61) |  |
|  | *Erysipelotrichi* | 100 | 0.50±0.77 | 83 | 0.17±0.18 | **0.27** |
|  |  |  | 0.15  (0.14, 0.34) |  | 0.14  (0.02, 0.28) |  |
|  | *Negativicutes* | 100 | 1.62±1.80 | 83 | 0.73±0.98 | **0.24** |
|  |  |  | 0.81  (0.38, 2.66) |  | 0.21  (0.11, 1.17) |  |
| *Bacteroidetes* | *Bacteroidia* | 100 | 2.42±3.37 | 94 | 0.90±0.87 | **0.35** |
|  |  |  | 0.97  (0.51, 2.30) |  | 0.72  (0.35, 1.10) |  |
|  | *Flavobacteriia* | 100 | 1.22±0.54 | 94 | 0.98±0.65 | **0.17** |
|  |  |  | 1.25  (1.09, 1.48) |  | 0.88  (0.66, 1.14) |  |
|  | *Shpingomonas* | 100 | 2.30±1.04 | 100 | 2.11±1.10 | **0.87** |
|  |  |  | 2.35  (2.00, 2.88) |  | 2.47  (1.58, 3.02) |  |
|  | *Cytophagia* | 83 | 0.08±0.05 | 72 | 0.08±0.10 | **0.74** |
|  |  |  | 0.10  (0.03, 0.12) |  | 0.04  (0.003, 0.14) |  |
| *Proteobacteria* | *Alphaproteobacteria* | 100 | 5.03±3.88 | 100 | 3.63±1.61 | 0.67 |
|  |  |  | 4.05  (3.67, 4.46) |  | 3.76  (2.89, 4.81) |  |
|  | *Betaproteobacteria* | 100 | 4.53±2.40 | 100 | 2.70±1.42 | 0.13 |
|  |  |  | 4.33  (2.47, 6.62) |  | 2.74  (1.84, 3.50) |  |
|  | *Gammaproteobacteria* | 100 | 19.35±11.99 | 100 | 26.18±27.62 | 0.92 |
|  |  |  | 20.81  (10.23, 27.91) |  | 14.50  (6.18, 34.38) |  |
| *Actinobacteria* | *Actinobacteria* | 100 | 9.83±4.83 | 100 | 7.68±4.58 | 0.37 |
|  |  |  | 8.91  (7.55, 13.13) |  | 8.00  (5.52, 9.32) |  |
|  | *Coriobacteria* | 100 | 0.52±0.64 | 67 | 0.16±0.16 | 0.14 |
|  |  |  | 0.20  (0.15, 0.63) |  | 0.11  (0, 0.31) |  |
|  | *Thermoleophilia* | 100 | 0.05±0.03 | 56 | 0.03±0.04 | 0.23 |
|  |  |  | 0.04  (0.02, 0.07) |  | 0.02  (0, 0.05) |  |
| *Fusobacteria* | *Fusobacteria* | 100 | 0.44±0.33 | 83 | 0.19±0.22 | **0.05** |
|  |  |  | 0.38  (0.22, 0.60) |  | 0.13  (0.04, 0.25) |  |
| *Cyanobacteria* | *Chloroplast* | 100 | 0.45±0.35 | 94 | 0.45±0.54 | 0.58 |
|  |  |  | 0.35  (0.24, 0.49) |  | 0.28  (0.08, 0.62) |  |
| *Verrucomicrobia* | *Verrucomicrobiae* | 83 | 0.10±0.13 | 83 | 0.13±0.25 | 0.84 |
|  |  |  | 0.07  (0.04, 0.10) |  | 0.06  (0.01, 0.15) |  |
|  | *Other* | 100 | 0.92±0.25 | 100 | 0.93±0.77 | 0.59 |
|  |  |  | 0.94  (0.86, 1.08) |  | 0.84  (0.60, 1.13) |  |

Supplementary Table S4. Percentages of positive samples and relative abundance (%) of different bacterial genera in microbial communities according to sequencing. In case of relative abundance, mean±SD and median (with 1st quartile, 3rd quartile) are presented. *p-value* shows the difference of relative abundance level between the coeliac disease and the control group.

* shows statistically significant difference in positive samples between the groups.

| **Class** | **Genus** | **Coeliac disease group** | | **Control group** | | **p-value** |
| --- | --- | --- | --- | --- | --- | --- |
|  |  | % of positive samples | Mean±SD | % of positive samples | Mean±SD |  |
|  |  |  | Median  (Q1, Q3) |  | Median  (Q1, Q3) |  |
| *Bacilli* | *Lactobacillus* | 100 | 13.02±10.37 | 100 | 29.95±26.06 | 0.45 |
|  |  |  | 10.38  (4.19, 21.44) |  | 34.00  (1.78, 55.62) |  |
|  | *Staphylococcus* | 100 | 11.27±16.91 | 100 | 5.69±4.43 | 0.92 |
|  |  |  | 4.99  (4.29, 6.29) |  | 4.93  (2.22, 6.90) |  |
|  | *Streptococccus* | 100 | 10.33±5.20 | 100 | 11.34±9.74 | 0.82 |
|  |  |  | 10.95  (8.46, 12.53) |  | 7.51  (3.73, 16.03) |  |
|  | *Lactococcus* | 100 | 0.25±0.17 | 89 | 0.16±0.12 | 0.29 |
|  |  |  | 0.20  (0.15, 0.26) |  | 0.15  (0.07, 0.26) |  |
|  | *Aerococcus* | 83 | 0.15±0.23 | 72 | 0.09±0.12 | 0.69 |
|  |  |  | 0.05  (0.03, 0.16) |  | 0.05  (0.003, 0.13) |  |
|  | *Other* | 100 | 1.83±0.89 | 100 | 1.23±0.92 | 0.22 |
|  |  |  | 1.97  (1.77, 2.06) |  | 0.96  (0.70, 1.71) |  |
| *Clostridia* | *Anaerococcus* | 100 | 1.96±3.71 | 89 | 0.18±0.17 | **0.006** |
|  |  |  | 0.48  (0.34, 0.72) |  | 0.15  (0.08, 0.23) |  |
|  | *Faecalibacterium* | 100 | 1.02±0.88 | 83 | 0.47±1.05 | 0.07 |
|  |  |  | 1.03  (0.32, 1.32) |  | 0.24  (0.06, 0.42) |  |
|  | *Eubacterium* | 100 | 2.30±3.78 | 89 | 0.32±0.32 | 0.07 |
|  |  |  | 0.68  (0.26, 1.87) |  | 0.25  (0.03, 0.59) |  |
|  | *Blautia* | 100 | 1.04±0.96 | 94 | 0.50±0.45 | 0.10 |
|  |  |  | 0.71  (0.49, 1.21) |  | 0.37  (0.20, 0.68) |  |
|  | *Ruminococcus* | 83 | 1.64±2.62 | 72 | 0.43±0.66 | 0.38 |
|  |  |  | 0.50  (0.06, 1.76) |  | 0.17  (0.01, 0.52) |  |
|  | *Dorea* | 100 | 0.41±0.60 | 78 | 0.17±0.26 | 0.17 |
|  |  |  | 0.13  (0.10, 0.40) |  | 0.08  (0.03, 0.18) |  |
|  | *Roseburia* | 100 | 0.56±0.68 | 89 | 0.25±0.37 | 0.18 |
|  |  |  | 0.25  (0.17, 0.69) |  | 0.12  (0.05, 0.28) |  |
|  | *Lachnoclostridium* | 83 | 0.41±0.61 | 78 | 0.23±0.29 | 0.40 |
|  |  |  | 0.20  (0.16, 0.24) |  | 0.12  (0.07, 0.25) |  |
|  | *Coprococcus* | 100 | 0.22±0.18 | 56 | 0.27±0.73 | 0.15 |
|  |  |  | 0.16  (0.08, 0.36) |  | 0.06  (0.00, 0.21) |  |
|  | *Finegoldia* | 100 | 0.07±0.03 | 72 | 0.08±0.10 | 0.71 |
|  |  |  | 0.06  (0.05, 0.08) |  | 0.05  (0.008, 0.11) |  |
|  | *Other* | 100 | 4.16±4.31 | 89 | 1.56±1.35 | 0.20 |
|  |  |  | 2.50  (1.29, 5.04) |  | 1.46  (0.46, 2.29) |  |
| *Negativicutes* | *Veillonella* | 100 | 1.54±1.76 | 72 | 0.59±0.98 | 0.08 |
|  |  |  | 0.70  (0.32, 2.54) |  | 0.12  (0.003, 1.00) |  |
|  | *Dialister* | 67 | 0.06±0.06 | 44 | 0.09±0.25 | 0.39 |
|  |  |  | 0.06  (0.008, 0.10) |  | 0.00  (0.00, 0.06) |  |
|  | *Other* | 67 | 0.03±0.04 | 33 | 0.05±0.08 | 0.53 |
|  |  |  | 0.02  (0.003, 0.04) |  | 0.00  (0.00, 0.06) |  |
| *Bacteroidia* | *Bacteroides* | 100 | 0.45±0.58 | 83 | 0.24±0.22 | 0.53 |
|  |  |  | 0.23  (0.10, 0.46) |  | 0.22  (0.07, 0.34) |  |
|  | *Alistipes* | 50 | 0.05±0.06 | 39 | 0.02±0.04 | 0.37 |
|  |  |  | 0.03  (0.00, 0.07) |  | 0.00  (0.00, 0.03) |  |
|  | *Porphyromonas* | 67 | 0.05±0.06 | 78 | 0.11±0.15 | 0.54 |
|  |  |  | 0.05  (0.01, 0.07) |  | 0.04  (0.01, 0.18) |  |
|  | *Other* | 100 | 1.87±3.30 | 94 | 0.53±0.76 | 0.19 |
|  |  |  | 0.56  (0.40, 0.80) |  | 0.25  (0.16, 0.51) |  |
| *Flavobacteriia* | *Flavobacterium* | 100 | 0.74±0.33 | 89 | 0.51±0.36 | 0.08 |
|  |  |  | 0.86  (0.64, 0.97) |  | 0.54  (0.32, 0.64) |  |
|  | *Chryseobacterium* | 83 | 0.26±0.31 | 94 | 0.32±0.63 | 0.97 |
|  |  |  | 0.17  (0.06, 0.29) |  | 0.14  (0.07, 0.28) |  |
|  | *Cloacibacterium* | 83 | 0.06±0.05 | 39 | 0.02±0.03 | 0.06 |
|  |  |  | 0.05  (0.03, 0.08) |  | 0.00  (0.00, 0.04) |  |
|  | *Other* | 100 | 0.17±0.07 | 83 | 0.13±0.20 | 0.06 |
|  |  |  | 0.18  (0.12, 0.23) |  | 0.07  (0.02, 0.17) |  |
| *Sphingobacteriia* | *Sediminibacterium* | 100 | 0.32±0.22 | 94 | 0.33±0.27 | 0.93 |
|  |  |  | 0.29  (0.20, 0.40) |  | 0.27  (0.15, 0.42) |  |
|  | *Hydrotalea* | 100 | 1.69±0.77 | 94 | 1.48±0.76 | 0.53 |
|  |  |  | 1.63  (1.48, 2.14) |  | 1.60  (1.21, 2.10) |  |
|  | *Other* | 100 | 0.29±0.22 | 100 | 0.30±0.26 | 1.0 |
|  |  |  | 0.23  (0.17, 0.37) |  | 0.27  (0.11, 0.42) |  |
| *Alphaproteobacteria* | *Bradyrhizobium* | 100 | 1.42±0.48 | 94 | 1.30±0.91 | 0.68 |
|  |  |  | 1.51  (1.47, 1.63) |  | 1.26  (0.71, 1.99) |  |
|  | *Phenylobacterium* | 100 | 0.37±0.33 | 89 | 0.26±0.30 | 0.42 |
|  |  |  | 0.23  (0.16, 0.52) |  | 0.19  (0.10, 0.27) |  |
|  | *Sphingomonas* | 67* | 1.62±3.51 | 17* | 0.02±0.06 | **0.01** |
|  |  |  | 0.13  (0.02, 0.56) |  | 0.00  (0.00, 0.00) |  |
|  | *Sphingobium* | 83 | 0.06±0.08 | 56 | 0.13±0.26 | 0.61 |
|  |  |  | 0.02  (0.01, 0.10) |  | 0.01  (0.00, 0.12) |  |
|  | *Other* | 100 | 1.56±0.56 | 100 | 1.94±1.36 | 0.35 |
|  |  |  | 1.76  (1.48, 1.89) |  | 1.54  (1.02, 2.89) |  |
| *Betaproteobacteria* | *Ralstonia* | 100 | 0.64±0.30 | 94 | 0.51±0.40 | 0.41 |
|  |  |  | 0.63  (0.54, 0.73) |  | 0.46  (0.23, 0.70) |  |
|  | *Neisseria* | 100 | 1.78±1.10 | 94 | 0.75±0.50 | 0.07 |
|  |  |  | 1.70  (1.08, 2.39) |  | 0.65  (0.43, 1.24) |  |
|  | *Other* | 100 | 2.12±1.97 | 100 | 1.44±0.96 | 0.45 |
|  |  |  | 1.41  (1.08, 2.53) |  | 1.32  (0.88, 1.76) |  |
| *Gammaproteobacteria* | *Moraxella* | 100 | 6.91±6.46 | 100 | 7.37±7.60 | 0.92 |
|  |  |  | 4.87  (2.14, 12.09) |  | 4.47  (1.98, 9.40) |  |
|  | *Buttiauxella* | 50 | 1.33±2.52 | 44 | 1.67±4.61 | 0.66 |
|  |  |  | 0.08  (0.00, 1.16) |  | 0.00  (0.00, 0.13) |  |
|  | *Acinetobacter* | 100 | 4.12±7.54 | 100 | 11.41±28.53 | 0.38 |
|  |  |  | 0.98  (0.63, 2.25) |  | 1.30  (1.03, 2.21) |  |
|  | *Enterobacter* | 83 | 3.20±5.35 | 94 | 1.56±2.58 | 0.59 |
|  |  |  | 1.28  (0.13, 2.56) |  | 0.14  (0.05, 1.75) |  |
|  | *Pseudomonas* | 100 | 0.64±0.30 | 100 | 0.98±1.98 | 0.53 |
|  |  |  | 0.64  (0.55, 0.82) |  | 0.46  (0.28, 0.79) |  |
|  | *Nevskia* | 83 | 0.06±0.05 | 67 | 0.04±0.05 | 0.44 |
|  |  |  | 0.06  (0.02, 0.08) |  | 0.02  (0.00, 0.08) |  |
|  | *Yersinia* | 67 | 0.07±0.08 | 78 | 0.12±0.15 | 0.57 |
|  |  |  | 0.05  (0.003, 0.12) |  | 0.07  (0.02, 0.18) |  |
|  | *Klebsiella* | 83 | 0.13±0.16 | 67 | 0.26±0.42 | 0.87 |
|  |  |  | 0.09  (0.02, 0.18) |  | 0.12  (0.00, 0.31) |  |
|  | *Other* | 100 | 2.73±1.82 | 100 | 2.77±3.51 | 0.48 |
|  |  |  | 2.72  (1.49, 3.57) |  | 1.26  (0.88, 2.70) |  |
| *Actinobacteria* | *Micrococcus* | 83 | 1.53±2.53 | 94 | 1.05±0.88 | 0.50 |
|  |  |  | 0.50  (0.20, 1.25) |  | 0.94  (0.37, 1.52) |  |
|  | *Bifidobacterium* | 100 | 0.42±0.41 | 89 | 0.21±0.17 | 0.26 |
|  |  |  | 0.32  (0.10, 0.68) |  | 0.21  (0.09, 0.28) |  |
|  | *Corynebacterium* | 100 | 2.75±1.17 | 100 | 2.81±1.86 | 0.93 |
|  |  |  | 2.82  (2.02, 3.72) |  | 2.64  (1.59, 4.07) |  |
|  | *Rothia* | 100 | 1.04±0.82 | 100 | 1.03±1.11 | 0.92 |
|  |  |  | 0.61  (0.47, 1.63) |  | 0.63  (0.44, 1.08) |  |
|  | *Actynomyces* | 100 | 1.84±2.0 | 94 | 0.29±0.28 | **0.006** |
|  |  |  | 0.87  (0.50, 2.75) |  | 0.22  (0.12, 0.34) |  |
|  | *Other* | 100 | 2.25±1.51 | 94 | 2.30±2.15 | 0.92 |
|  |  |  | 1.69  (1.16, 2.97) |  | 1.77  (1.30, 2.71) |  |
| *Fusobacteria* | *Fusobacterium* | 50 | 0.05±0.07 | 50 | 0.08±0.16 | 0.83 |
|  |  |  | 0.03  (0, 0.06) |  | 0.02  (0, 0.10) |  |
|  | *Leptotrichia* | 100 | 0.40±0.29 | 72 | 0.10±0.12 | **0.005** |
|  |  |  | 0.36  (0.24, 0.44) |  | 0.06  (0.004, 0.14) |  |
| *Verrucomicrobiae* | *Akkermansia* | 83* | 0.09±0.12 | 28* | 0.02±0.03 | **0.01** |
|  |  |  | 0.04  (0.03, 0.09) |  | 0.00  (0.00, 0.02) |  |
|  | *Other* | 50 | 0.01±0.02 | 78 | 0.06±0.06 | 0.07 |
|  |  |  | 0.0005  (0.00, 0.02) |  | 0.03  (0.01, 0.13) |  |

Supplementary Table S5. Percentages of positive samples and relative abundance (%) of *Lactobacillus*, *Bifidobacterium*, *Akkermansia* and *Faecalibacterium* species in microbial communities according to sequencing. In case of relative abundance, mean±SD and median (with 1st quartile, 3rd quartile) are presented. *p-value* shows the difference of relative abundance level between the coeliac disease and the control group.

| **Genus** | **Species** | **Coeliac disease group** | | **Control group** | | **p-value** |
| --- | --- | --- | --- | --- | --- | --- |
|  |  | % of positive samples | Mean±SD | % of positive samples | Mean±SD |  |
|  |  |  | Median  (Q1, Q3) |  | Median  (Q1, Q3) |  |
| *Lactobacillus* | *L. plantarum* | 50 | 1.50±2.66 | 56 | 5.74±11.91 | **0.65** |
|  |  |  | 0.09  (0.00, 1.67) |  | 0.26  (0.00, 5.79) |  |
|  | *L. curvatus* | 17 | 0.02±0.05 | 17 | 0.04±0.12 | 1.00 |
|  |  |  | 0.00  (0.00, 0.00) |  | 0.00  (0.00, 0.00) |  |
|  | *L. iners* | 33 | 0.43±0.99 | 44 | 0.39±1.26 | 0.91 |
|  |  |  | 0.00  (0.00, 0.11) |  | 0.00  (0.00, 0.08) |  |
|  | *L. mucosae* | 17 | 0.002±0.006 | 0 | 0.0±0.0 | **0.10** |
|  |  |  | 0.00  (0.00, 0.00) |  | 0.00  (0.00, 0.00) |  |
|  | *L. casei* | 67 | 3.09±4.43 | 56 | 15.28±20.40 | **0.65** |
|  |  |  | 0.52  (0.08, 6.01) |  | 0.41  (0.00, 27.48) |  |
|  | *L. fermentum* | 33 | 1.04±2.10 | 28 | 1.45±3.06 | **0.058** |
|  |  |  | 0.00  (0.00, 0.77) |  | 0.00  (0.00, 0.05) |  |
|  | *L. salivarius* | 83 | 0.66±0.71 | 78 | 0.44±0.74 | **0.40** |
|  |  |  | 0.42  (0.11, 1.10) |  | 0.22  (0.03, 0.46) |  |
|  | *L. reuteri* | 17 | 0.005±0.01 | 6 | 0.03±0.14 | **0.49** |
|  |  |  | 0.00  (0.00, 0.00) |  | 0.00  (0.00, 0.00) |  |
|  | *L. rhamnosus* | 0 | 0.0±0.0 | 11 | 0.03±0.11 | **0.44** |
|  |  |  | 0.00  (0.00, 0.00) |  | 0.00  (0.00, 0.00) |  |
|  | *L. crispatus* | 0 | 0.0±0.0 | 28 | 0.05±0.10 | 0.16 |
|  |  |  | 0.00  (0.00, 0.00) |  | 0.00  (0.00, 0.09) |  |
|  | *L. hominis* | 0 | 0.0±0.0 | 22 | 0.01±0.03 | 0.24 |
|  |  |  | 0.00  (0.00, 0.00) |  | 0.00  (0.00, 0.00) |  |
|  | *L. sakei* | 0 | 0.0±0.0 | 11 | 0.04±0.13 | 0.44 |
|  |  |  | 0.00  (0.00, 0.00) |  | 0.00  (0.00, 0.00) |  |
|  | *L. zeae* | 0 | 0.0±0.0 | 12 | 0.01±0.03 | 0.43 |
|  |  |  | 0.00  (0.00, 0.00) |  | 0.00  (0.00, 0.00) |  |
|  | *L. jensenii* | 0 | 0.0±0.0 | 6 | 0.0009±0.003 | 0.63 |
|  |  |  | 0.00  (0.00, 0.00) |  | 0.00  (0.00, 0.00) |  |
|  | *L. senmaizukei* | 17 | 0.003±0.008 | 0 | 0.0±0.0 | 0.10 |
|  |  |  | 0.00  (0.00, 0.00) |  | 0.00  (0.00, 0.00) |  |
|  | *L. paracasei* | 0 | 0.0±0.0 | 11 | 1.22±5.11 | 0.44 |
|  |  |  | 0.00  (0.00, 0.00) |  | 0.00  (0.00, 0.00) |  |
|  | *L. oligofermnetas* | 0 | 0.0±0.0 | 6 | 0.0009±0.004 | 0.63 |
|  |  |  | 0.00  (0.00, 0.00) |  | 0.00  (0.00, 0.00) |  |
|  | *L. gasseri* | 0 | 0.0±0.0 | 6 | 0.0007±0.003 | 0.63 |
|  |  |  | 0.00  (0.00, 0.00) |  | 0.00  (0.00, 0.00) |  |
|  | *L. acidophilus* | 0 | 0.0±0.0 | 6 | 0.006±0.02 | 0.63 |
|  |  |  | 0.00  (0.00, 0.00) |  | 0.00  (0.00, 0.00) |  |
| *Bifidobacterium* | *B. bifidum* | 17 | 0.003±0.008 | 17 | 0.005±0.017 | 1.0 |
|  |  |  | 0.00  (0.00, 0.00) |  | 0.00  (0.00, 0.00) |  |
|  | *B. animalis* | 50 | 0.04±0.06 | 50 | 0.05±0.09 | 1.0 |
|  |  |  | 0.01  (0.00, 0.04) |  | 0.008  (0.00, 0.08) |  |
|  | *B. adolescentis* | 0 | 0.0±0.0 | 6 | 0.009±0.04 | 0.63 |
|  |  |  | 0.00  (0.00, 0.00) |  | 0.00  (0.00, 0.00) |  |
|  | *B. longum* | 0 | 0.0±0.0 | 6 | 0.004±0.02 | 0.63 |
|  |  |  | 0.00  (0.00, 0.00) |  | 0.00  (0.00, 0.00) |  |
| *Akker-mansia* | *A. muciniphila* | 50^*^ | 0.01±0.03 | 28^*^ | 0.07±0.11 | 0.20 |
|  |  |  | 0.02  (0.00, 0.15) |  | 0.00  (0.00, 0.02) |  |
| *Faecali-bacterium* | *F. prausnitzii* | 100 | 0.88±0.7 | 83 | 0.36±0.7 | **0.049** |
|  |  |  | 0.86  (0.29, 1.09) |  | 0.21  (0.03, 0.38) |  |
| *Anaero-coccus* | *A. hydrogenalis* | 50^*^ | 0.02±0.03 | 11^*^ | 0.004±0.01 | **0.04** |
|  |  |  | 0.008  (0.00, 0.07) |  | 0.00  (0.00, 0.00) |  |
|  | *A. octavius* | 100 | 0.07±1.4 | 72 | 0.015±0.34 | **0.01** |
|  |  |  | 0.13  (0.05, 0.24) |  | 0.00  (0.00, 0.01) |  |
| *Actino*  *-myces* | *A. odontolyticus* | 100 | 0.73±1.15 | 66 | 0.11±0.23 | **0.03** |
|  |  |  | 0.19  (0.13, 0.81) |  | 0.04  (0.00,0.11) |  |

Supplementary Table S6. Correlation coefficients between bacterial phyla, classes, and immune markers (Spearman’s rank-order correlation).

| **Phylum** | **TGF-β1** | **TGF-β2** | **sIgA** | **MFG-E8** | **sCD14** |
| --- | --- | --- | --- | --- | --- |
| *Firmicutes* | 0.157 | 0.330 | -0.043 | -0.332 | 0.067 |
| *Bacteroidetes* | -0.067 | -0.174 | 0.025 | 0.276 | 0.117 |
| *Proteobacteria* | -0.179 | -0.355 | 0.045 | 0.350 | -0.029 |
| *Actinobacteria* | 0.220 | 0.089 | 0.099 | 0.037 | 0.088 |
| *Fusobacteria* | -0.017 | 0.055 | -0.103 | -0.185 | 0.018 |
| *Cyanobacteria* | -0.101 | -0.301 | 0.008 | -0.154 | -0.120 |
| *Verrucomicrobia* | 0.239 | 0.207 | 0.220 | 0.143 | 0.270 |
| **Class** |  |  |  |  |  |
| *Bacilli* | 0.115 | 0.216 | -0.146 | -0.401 | -0.030 |
| *Clostridia* | 0.061 | 0.108 | 0.150 | -0.090 | 0.012 |
| *Erysipelotrichi* | 0.258 | 0.205 | 0.323 | 0.153 | 0.391 |
| *Negativicutes* | -0.183 | -0.040 | -0.032 | 0.190 | 0.314 |
| *Bacteroidia* | 0.140 | 0.123 | 0.113 | 0.281 | 0.287 |
| *Flavobacteriia* | -0.217 | -0.365 | -0.048 | **0.454** | 0.225 |
| *Shpingomonas* | -0.122 | -0.278 | -0.093 | -0.100 | -0.154 |
| *Cytophagia* | -0.152 | -0.315 | -0.241 | -0.282 | -0.241 |
| *Alphaproteobacteria* | -0.272 | -0.381 | -0.239 | 0.142 | -0.108 |
| *Betaproteobacteria* | -0.091 | -0.163 | 0.088 | 0.059 | 0.159 |
| *Gammaproteobacteria* | -0.072 | -0.254 | 0.148 | 0.346 | 0.019 |
| *Actinobacteria* | 0.243 | 0.057 | 0.062 | 0.027 | 0.047 |
| *Coriobacteria* | -0.008 | 0.094 | -0.195 | -0.323 | -0.181 |
| *Thermoleophilia* | -0.076 | -0.108 | -0.210 | 0.048 | -0.394 |
| *Fusobacteria* | -0.011 | 0.061 | -0.100 | -0.179 | 0.024 |
| *Chloroplast* | -0.101 | -0.297 | -0.0004 | -0.148 | -0.128 |
| *Verrucomicrobiae* | 0.324 | 0.275 | 0.327 | 0.097 | 0.273 |

Supplementary Table S7. Correlation coefficients between bacterial genera and immune markers (Spearman’s rank-order correlation; bold indicates *p-value* <0.05).

| **Genus** | **TGF-β1** | **TGF-β2** | **sIgA** | **MFG-E8** | **sCD14** |
| --- | --- | --- | --- | --- | --- |
| *Lactobacillus* | 0.031 | 0.091 | -0.185 | -0.257 | -0.089 |
| *Staphylococcus* | 0.227 | 0.204 | 0.144 | -0.357 | 0.024 |
| *Streptococccus* | 0.041 | 0.135 | 0.118 | -0.090 | 0.333 |
| *Lactococcus* | -0.248 | -0.360 | -0.188 | -0.136 | 0.065 |
| *Aerococcus* | 0.164 | -0.041 | -0.148 | -0.075 | -0.026 |
| *Anaerococcus* | -0.047 | -0.099 | 0.012 | -0.045 | -0.064 |
| *Faecalibacterium* | 0.009 | 0.078 | 0.076 | 0.077 | 0.083 |
| *Eubacterium* | 0.072 | 0.061 | 0.216 | 0.091 | 0.095 |
| *Blautia* | -0.065 | -0.060 | -0.019 | -0.023 | -0.123 |
| *Ruminococcus* | 0.127 | 0.084 | 0.172 | 0.114 | 0.168 |
| *Dorea* | -0.079 | -0.055 | -0.056 | -0.248 | -0.217 |
| *Roseburia* | 0.113 | 0.170 | 0.040 | -0.173 | -0.085 |
| *Lachnoclostridium* | 0.133 | -0.110 | -0.080 | -0.226 | -0.162 |
| *Coprococcus* | 0.168 | 0.239 | 0.088 | -0.143 | 0.020 |
| *Finegoldia* | 0.045 | -0.123 | 0.021 | -0.050 | 0.082 |
| *Veillonella* | -0.235 | -0.008 | -0.005 | 0.226 | 0.398 |
| *Dialister* | -0.059 | -0.0005 | -0.165 | 0.032 | -0.005 |
| *Bacteroides* | 0.102 | -0.114 | -0.020 | 0.064 | -0.021 |
| *Alistipes* | 0.002 | -0.227 | -0.053 | 0.185 | -0.210 |
| *Porphyromonas* | 0.115 | 0.098 | 0.095 | 0.168 | 0.388 |
| *Fusobacterium* | -0.127 | 0.085 | -0.108 | 0.125 | -0.174 |
| *Lepotrichia* | 0.312 | -0.041 | 0.367 | 0.056 | 0.160 |
| *Flavobacterium* | -0.168 | -0.302 | 0.120 | 0.253 | 0.274 |
| *Chryseobacterium* | -0.133 | **-0.419** | -0.186 | 0.227 | 0.107 |
| *Cloacibacterium* | 0.027 | 0.160 | -0.006 | 0.047 | -0.030 |
| *Sediminibacterium* | -0.029 | -0.136 | 0.144 | 0.111 | 0.036 |
| *Hydrotalea* | -0.078 | -0.304 | -0.150 | -0.089 | -0.136 |
| *Bradyrhizobium* | 0.040 | -0.120 | 0.107 | 0.134 | 0.084 |
| *Phenylobacterium* | 0.206 | 0.269 | 0.002 | -0.005 | 0.092 |
| *Sphingomonas* | -0.042 | -0.257 | -0.135 | -0.006 | -0.267 |
| *Sphingobium* | -0.372 | **-0.513** | -0.208 | -0.089 | -0.090 |
| *Ralstonia* | -0.038 | -0.207 | 0.068 | 0.066 | 0.013 |
| *Neisseria* | 0.010 | 0.157 | 0.192 | 0.013 | 0.309 |
| *Moraxella* | 0.202 | 0.077 | 0.223 | 0.145 | 0.209 |
| *Buttiauxella* | 0.056 | 0.021 | 0.279 | 0.393 | 0.326 |
| *Acinetobacter* | -0.058 | -0.150 | -0.048 | -0.078 | -0.165 |
| *Enterobacter* | 0.037 | -0.218 | -0.021 | 0.221 | 0.105 |
| *Pseudomonas* | 0.069 | -0.047 | -0.150 | -0.229 | -0.281 |
| *Nevskia* | -0.135 | -0.144 | -0.348 | -0.143 | -0.354 |
| *Yersinia* | 0.179 | -0.021 | 0.223 | 0.164 | 0.245 |
| *Klebsiella* | -0.196 | -0.213 | -0.006 | 0.054 | 0.083 |
| *Micrococcus* | 0.259 | -0.079 | -0.123 | -0.081 | -0.097 |
| *Bifidobacterium* | 0.032 | 0.199 | 0.015 | 0.127 | 0.083 |
| *Corynebacterium* | 0.146 | -0.146 | -0.057 | -0.058 | -0.210 |
| *Rothia* | 0.019 | 0.074 | -0.106 | -0.091 | 0.157 |
| *Actynomyces* | -0.260 | -0.183 | -0.224 | -0.024 | 0.095 |
| *Akkermansia* | 0.209 | 0.363 | 0.095 | -0.016 | 0.146 |

Supplementary Table S8. Correlation coefficents between *Lactobacillus*, *Bifidobacterium*, *Akkermansia* and *Faecalibacterium* species and immune markers (Spearman’s rank-order correlation; bold indicates *p-value* <0.05).

| **Species** | **TGF-β1** | **TGF-β2** | **sIgA** | **MFG-E8** | **sCD14** |
| --- | --- | --- | --- | --- | --- |
| *L. plantarum* | -0.110 | 0.072 | 0.041 | -0.246 | 0.132 |
| *L. curvatus* | -0.297 | -0.345 | -0.201 | 0.015 | -0.076 |
| *L. iners* | 0.156 | -0.173 | -0.127 | 0.113 | 0.055 |
| *L. mucosae* | -0.317 | -0.316 | -0.302 | 0.196 | -0.016 |
| *L. casei* | 0.079 | 0.171 | -0.049 | -0.037 | 0.052 |
| *L. fermentum* | -0.043 | -0.071 | 0.084 | 0.196 | -0.018 |
| *L. salivarius* | 0.189 | 0.049 | -0.166 | -0.311 | -0.045 |
| *L. reuteri* | 0.099 | 0.349 | 0.395 | 0.149 | **0.431** |
| *L. rhamnosus* | 0.094 | -0.201 | 0.146 | 0.097 | 0.072 |
| *L. crispatus* | -0.019 | -0.237 | -0.024 | -0.008 | -0.118 |
| *L. hominis* | -0.367 | -0.338 | -0.164 | -0.039 | 0.025 |
| *L. sakei* | 0.221 | 0.027 | 0.098 | 0.209 | -0.046 |
| *L. zeae* | -0.198 | -0.133 | -0.198 | -0.283 | -0.187 |
| *L. jensenii* | 0.286 | 0.166 | -0.211 | -0.015 | -0.218 |
| *L. senmaizukei* | -0.317 | -0.136 | -0.076 | -0.075 | -0.219 |
| *L. paracasei* | -0.170 | -0.393 | -0.113 | -0.052 | -0.316 |
| *L. oligofermnetas* | 0.286 | 0.166 | -0.211 | -0.015 | -0.219 |
| *L. gasseri* | 0.075 | 0.196 | 0.030 | -0.136 | -0.047 |
| *L. acidophilus* | 0.317 | 0.316 | 0.287 | 0.317 | 0.203 |
| *B. bifidum* | -0.344 | -0.323 | -0.315 | 0.207 | 0.021 |
| *B. animalis* | -0.098 | -0.078 | -0.342 | **-0.435** | **-0.472** |
| *B. adolescentis* | -0.226 | 0.015 | -0.045 | -0.226 | 0.047 |
| *B. longum* | 0.075 | 0.196 | 0.030 | -0.136 | -0.047 |
| Unclassified | 0.217 | 0.212 | 0.292 | 0.328 | 0.300 |
| *A. muciniphila* | **0.430** | **0.536** | 0.241 | -0.057 | 0.237 |
| *F. prausnitzii* | 0.048 | 0.109 | 0.050 | 0.076 | 0.074 |

Supplementary Table S9. *Bifidobacterium* and *Lactobacillus* sp. and their counts in breast milk with genetic risk of coeliac diseases (CD) and healthy (H) analysed by PCR-DGGE and real-time PCR.

| **Sample ID** | ***Bifidobacterium* sp.** | | ***Lactobacillus* sp.** | | |
| --- | --- | --- | --- | --- | --- |
|  | PCR-DGGE | Real-time PCR  (log10plasmid gene copies/ml) | | PCR-DGGE | Real-time PCR  (log10plasmid gene copies/ml) |
| CD1 | *B. bifidum* | 1.8 | | *L. paracasei*  *L. plantarum*  *L. iners* | 1.54 |
| CD2 | *B. adolescentis* | 0.9 | | *L. casei*  *L. paracasei*  *L. plantarum*  *L. salivarius* | 1.59 |
| CD3 | *B. adolescentis* | 1.6 | | *L. casei*  *L. paracasei*  *L. plantarum*  *L. salivarius* | 1.77 |
| CD4 | *B.pseudo-*  *catenulatum*  *B. adolescentis* | 2.1 | | *L. casei*  *L. paracasei*  *L. plantarum*  *Allacoiccus otitis*  *L. salivarius* | 2.15 |
| CD5 | *B. adolescentis* | 2.25 | | *L. casei*  *L. paracasei*  *L. plantarum*  *L. salivarius* | 2.29 |
| CD6 | *B. infantis* | 0.82 | | *L. plantarum*  *L. paracasei*  *L. casei*  *L. iners* | 3.05 |
| CG1 | *B. adolescentis*  *B. bifidum* | 1.55 | | *L. plantarum*  *L. casei*  *L. paracasei* | 2.54 |
| CG2 | *B. adolescentis* | 2.1 | | *L. casei*  *L. paracasei*  *L. plantarum* | 2.17 |
| CG3 | *B. longum* subsp*. longum* | 1.5 | | *L. casei*  *L. paracasei*  *L. plantarum* | 2.20 |
| CG4 | *B. adolescentis* | 1.13 | | *L. casei*  *L. zeae*  *L. paracasei*  *L. crispatus*  *L. salivarius*  *L. iners* | 3.24 |
| CG5 | *B. adolescentis* | 1.3 | | *L. plantarum*  *L. casei*  *L. paracasei*  *L. iners* | 2.99 |
| CG6 | *B. longum* subsp*. longum*  *B. animalis sbsp. lactis* | 1.62 | | *L. paracasei*  *L. plantarum*  *L. iners*  *L. salivarius* | 3.38 |
| CG7 | *B. adolescentis* | 1.57 | | *L. paracasei*  *L. casei*  *L. plantarum*  *L. salivarius* | 3.01 |
| CG8 | *B. adolescentis* | 1.83 | | *L. casei*  *L. paracasei*  *L. plantarum*  *L. iners*  *L. saliavrius* | 2.61 |
| CG9 | ND | ND | | *L. plantarum*  *L. casei*  *L. paracasei* | 3.11 |
| CG10 | *B. animalis sbsp. lactis* | 1.26 | | *L. paracasei*  *L. iners*  *L. salivarius*  *L. iners* | 3.02 |
| CG11 | *B. adolescentis* | 1.52 | | *L.plantarum*  *L. casei*  *L. paracasei*  *L. salivarius* | 2.50 |
| CG12 | *B. adolescentis* | 1.7 | | *L. johhonsonii*  *L. gasseri*  *L. salivarius*  *L. paracasei subsp.*  *L. paracasei*  *L. casei* | 2.56 |
| CG13 | *B. adolescentis* | ND | | *L. plantarum*  *L. salivarius*  *L. casei* | 1.44 |
| CG14 | *B. bifidum*  *B. animalis* | 1.3 | | *L. casei*  *L. crispatus*  *L. plantarum*  *L. paracasei* | 2.19 |
| CG15 | *B. longum* subsp*. longum* | 1.1 | | *L. paracasei*  *L. plantarum*  *L. salivarius*  *L. gasseri* | 3.66 |
| CG16 | *B. adolescentis*  *B. breve*  *B. animalis sbsp. lactis* | 1.8 | | *L. plantarum*  *L. paracasei*  *L. casei*  *L. iners* | 3.74 |
| CG17 | *B. adolescentis* | 1.0 | | *L. plantarum*  *L. paracasei*  *L. salivarius* | 2.51 |
| CG18 | ND | 0.9 | | *L. plantarum*  *L. salivarius*  *Weisella ghanensis*  *L. paracasei*  *L. iners* | 3.28 |
