# Supplementary material for: Maternal breast milk microbiota and immune markers in relation to subsequent development of celiac disease in offspring
Source: Sci Rep. 2022 Apr 22;12:6607. doi: 10.1038/s41598-022-10679-x (PMC9033794; doi:10.1038/s41598-022-10679-x)
Supplement: Supplementary file 2 — Supplementary Figure S1. [file 41598_2022_10679_MOESM2_ESM.pdf]

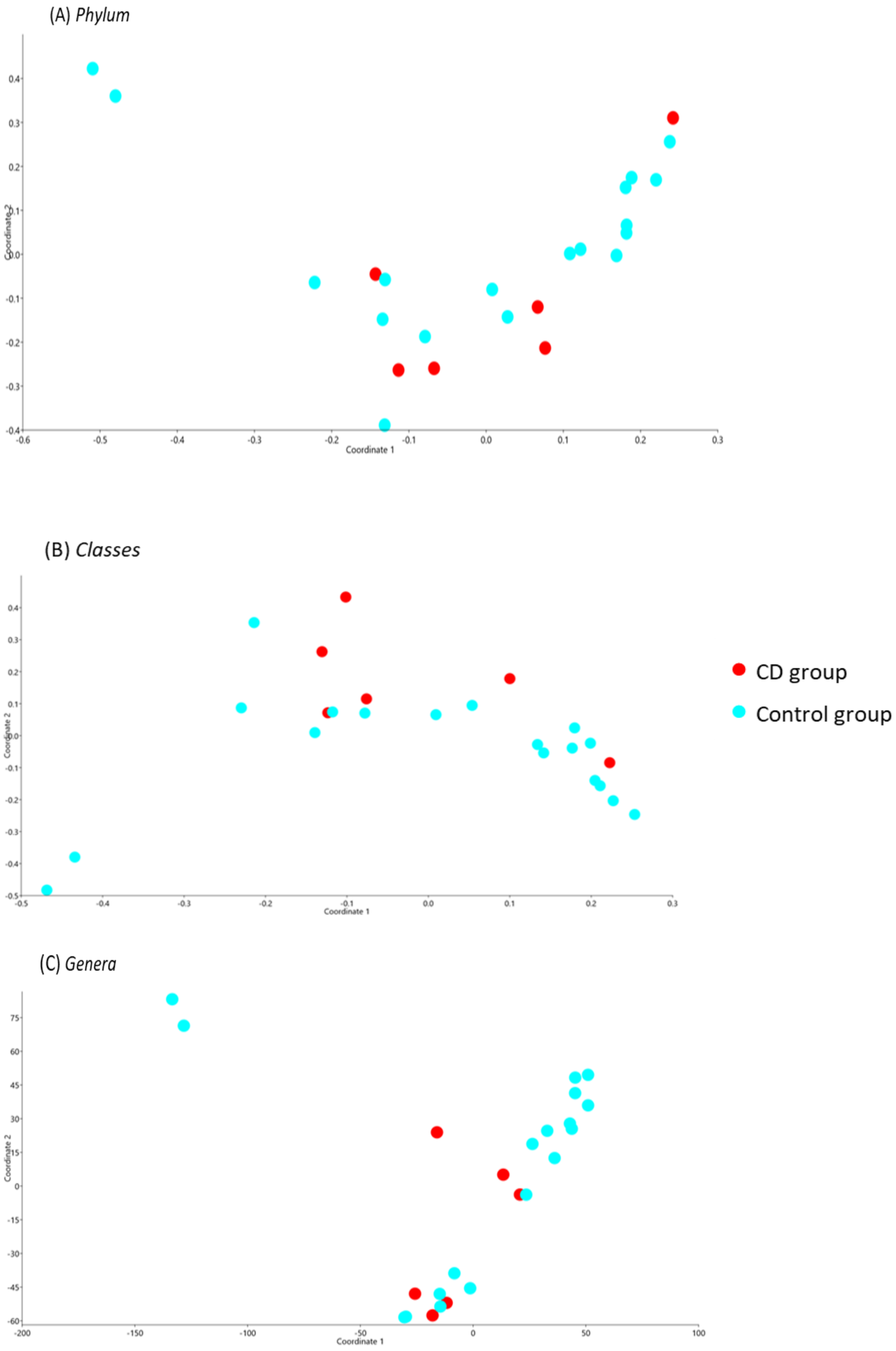

(A) *Phylum*
Coordinate 1
Coordinate 2
(B) *Classes*
Coordinate 1
Coordinate 2
CD group
Control group
(C) *Genera*
Coordinate 1
Coordinate 2

Figure S1. Principal coordinate analysis (PCoA) of bacterial communities in the samples analyzed based on (A) phyla, (B) classes and (C) genera levels. A principal coordinate analysis plot demonstrates different clustering of different specimens when comparing the breastmilk samples in the CD group to those in the control group.
